# Effects of Melatonin Formulations on Caffeine-Evoked Cortical Activity and Pentobarbital-Induced Sleep in Mice

**DOI:** 10.3390/ijms27156906

**Published:** 2026-08-01

**Authors:** Nurhan Sahin, Mehmet Yabas, Besir Er, Fusun Erten, Oznur Ece Durmaz Kursun, Ismail Gurkan Cikim, Berkan Kaplan, Gozde Erek, Busra Ozmen, Cemal Orhan, Ertugrul Kilic, Kazim Sahin

**Affiliations:** 1 Department of Animal Nutrition, Faculty of Veterinary Medicine, Firat University, Elazig 23119, Türkiye; gerek@firat.edu.tr (G.E.); busraagzikucuk01@gmail.com (B.O.); corhan@firat.edu.tr (C.O.); nsahinkm@yahoo.com (K.S.); 2 Department of Immunology, Faculty of Medicine, Malatya Turgut Ozal University, Malatya 44210, Türkiye; mehmetyabas@ozal.edu.tr; 3 Department of Medical Services and Techniques, Vocational School of Health Services, Firat University, Elazig 23119, Türkiye; ber@firat.edu.tr; 4 Department of Veterinary Science, Pertek Sakine Genç Vocational School, Munzur University, Tunceli 62500, Türkiye; fusunerten@munzur.edu.tr; 5 Department of Pharmacology, Faculty of Pharmacy, Firat University, Elazig 23119, Türkiye; oedkursun@firat.edu.tr; 6 Department of Biochemistry, Faculty of Medicine, Kahramanmaras Sutcu Imam University, Kahramanmaras 46040, Türkiye; ismailgurkancikim@ksu.edu.tr; 7 Department of Neurology, Servet Unsal Medical Center, Ankara 06200, Türkiye; kaplanberkan@yahoo.com; 8 Department of Physiology, Faculty of Medicine, Istanbul Medeniyet University, Istanbul 34700, Türkiye; ertugrul.kilic@medeniyet.edu.tr

**Keywords:** melatonin, lipid-multiparticulate formulation, cortical activity, electrocorticography, sedative-hypnotic activity, oxidative stress, neuroinflammation

## Abstract

Melatonin has chronobiotic, antioxidant, anti-inflammatory, and neuromodulatory properties, and its oral effects may vary according to formulation. This study compared conventional melatonin with a lipid-multiparticulate (LMP) formulation in two acute pharmacological mouse paradigms. Eighty-four male BALB/c mice were assigned to caffeine-evoked cortical activity or pentobarbital-induced sedative-hypnotic experiments. Mice received caffeine (7.5 mg/kg, i.p.) alone or with standard or LMP-melatonin (10 or 20 mg/kg, oral). ECoG recorded cortical electrical activity under urethane anaesthesia. In a separate cohort, latency to and duration of pentobarbital-induced loss of the righting reflex were measured. Circulating neurochemical and redox markers, as well as neurotransmitter- and inflammation-related gene and protein expression, were evaluated. Caffeine increased spike frequency and altered ECoG amplitude, together with unfavorable changes in circulating neurochemical and redox markers and in neurotransmitter receptor- and inflammation-related outcomes. Both melatonin formulations attenuated several of these changes in a dose-dependent manner, with the strongest effects generally observed in the high-dose LMP-melatonin group. Compared with conventional melatonin, LMP-melatonin produced greater modulation of ECoG activity, circulating biochemical markers, neurotransmitter-receptor expression, and IL-6 and TNF-α-related outcomes. LMP-melatonin also produced a greater reduction in the latency to and a greater prolongation of the duration of pentobarbital-induced loss of the righting reflex. LMP-melatonin produced greater formulation-dependent effects than conventional melatonin on caffeine-evoked cortical electrical activity, pentobarbital-induced sedative-hypnotic responses, and selected biochemical and molecular outcomes. However, pharmacokinetic exposure, physiological sleep stages, sleep architecture, sleep quality, and direct measures of neuronal injury were not assessed. Therefore, the findings do not directly establish enhanced bioavailability, physiological sleep promotion, or neuroprotection.

## 1. Introduction

Sleep is a tightly regulated neurobiological state determined by circadian timing and homeostatic drive and is essential for cognitive performance, emotional stability, metabolic homeostasis, and immune competence [1,2,3]. Chronic sleep deprivation and fragmentation, now rampant, have mechanistically been associated with increased oxidative stress, microglial activation, neuroinflammation, and compromised synaptic plasticity, thus increasing vulnerability to mood disorders, cognitive decline, and systemic dysfunction [3,4,5]. Conventional hypnotics provide primarily short-term symptomatic relief and may be associated with cognitive, psychomotor, and residual daytime adverse effects, highlighting the need for treatment approaches that restore physiologic sleep processes rather than simply inducing sedation [6,7]. The sleep–wake control arises from interactions of excitatory and inhibitory activity within widely spread cortical and subcortical circuits. GABAergic signaling facilitates the onset and maintenance of sleep, while glutamatergic activity underlies states that maintain arousal and synaptic plasticity; serotonergic projections from the brainstem temporally integrate these systems on a context-dependent basis across the cortex, hippocampus, and hypothalamus [8,9,10,11]. In tandem with redox disequilibrium and inflammatory activation, sleep loss disrupts this balance, suggesting that both key features of sleep restoration (and/or stability) may be required simultaneously to restore neurotransmitter networks and oxidative-immune homeostasis properly [12,13].

Melatonin, a pineal indoleamine with also peripheral synthesis, regulates circadian timing and acts as a pleiotropic redox-active molecule [8,10,14]. In addition to the direct radical scavenging described above, melatonin also upregulates endogenous antioxidant defenses and downregulates inflammatory signaling, thereby preserving mitochondrial function and synaptic integrity under stress conditions [12,15]. Indeed, in experimental sleep destruction, melatonin decreases the coupling between oxidative stress and neuroinflammation and improves behavioral performance, suggesting that it may act as a neuroprotective modulator of sleep [13,16]. BALB/c mice are widely regarded as a melatonin-deficient or markedly low melatonin-producing strain because pineal melatonin concentrations and melatonin-synthesizing enzyme activities are often extremely low or undetectable [17,18,19,20]. Nevertheless, sensitive HPLC-based studies have detected low-amplitude and transient melatonin rhythms in this strain, suggesting markedly reduced rather than completely absent melatonin production [21,22]. This strain-specific characteristic should therefore be considered when interpreting endogenous circulating melatonin concentrations, particularly during the light phase.

Formulation-dependent differences may influence oral melatonin efficacy in dissolution, absorption, and systemic exposure. Lipid-multiparticulate (LMP) delivery systems are designed to modify drug release and improve the oral absorption of poorly soluble compounds [23]. However, whether the LMP formulation alters systemic or central melatonin exposure was not directly examined in the present study. Therefore, standard and LMP-melatonin were compared using complementary electrophysiological, sedative-hypnotic, biochemical, and molecular outcomes in a caffeine-evoked arousal model [24,25]. Cortical electrical activity was assessed by ECoG under urethane anaesthesia, together with circulating neurochemical and redox markers and region-specific expression of neurotransmitter receptor- and inflammation-related targets in the cortex, hippocampus, and hypothalamus. Sedative-hypnotic responsiveness was assessed separately using pentobarbital-induced loss of the righting reflex. Potential formulation-dependent pharmacokinetic differences were considered only as literature-supported explanations and not as mechanisms established by the present experiments. We hypothesized that LMP-melatonin would produce greater modulation of caffeine-evoked cortical electrical activity, greater enhancement of pentobarbital-induced sedative-hypnotic responses, and more favorable changes in circulating neurochemical and redox markers, as well as in neurotransmitter- and inflammation-related molecular outcomes, than conventional melatonin.

## 2. Results

### 2.1. Effects of Caffeine and Melatonin Formulations on Caffeine-Evoked Cortical Electrical Activity Under Urethane Anesthesia

**Figure 1:**
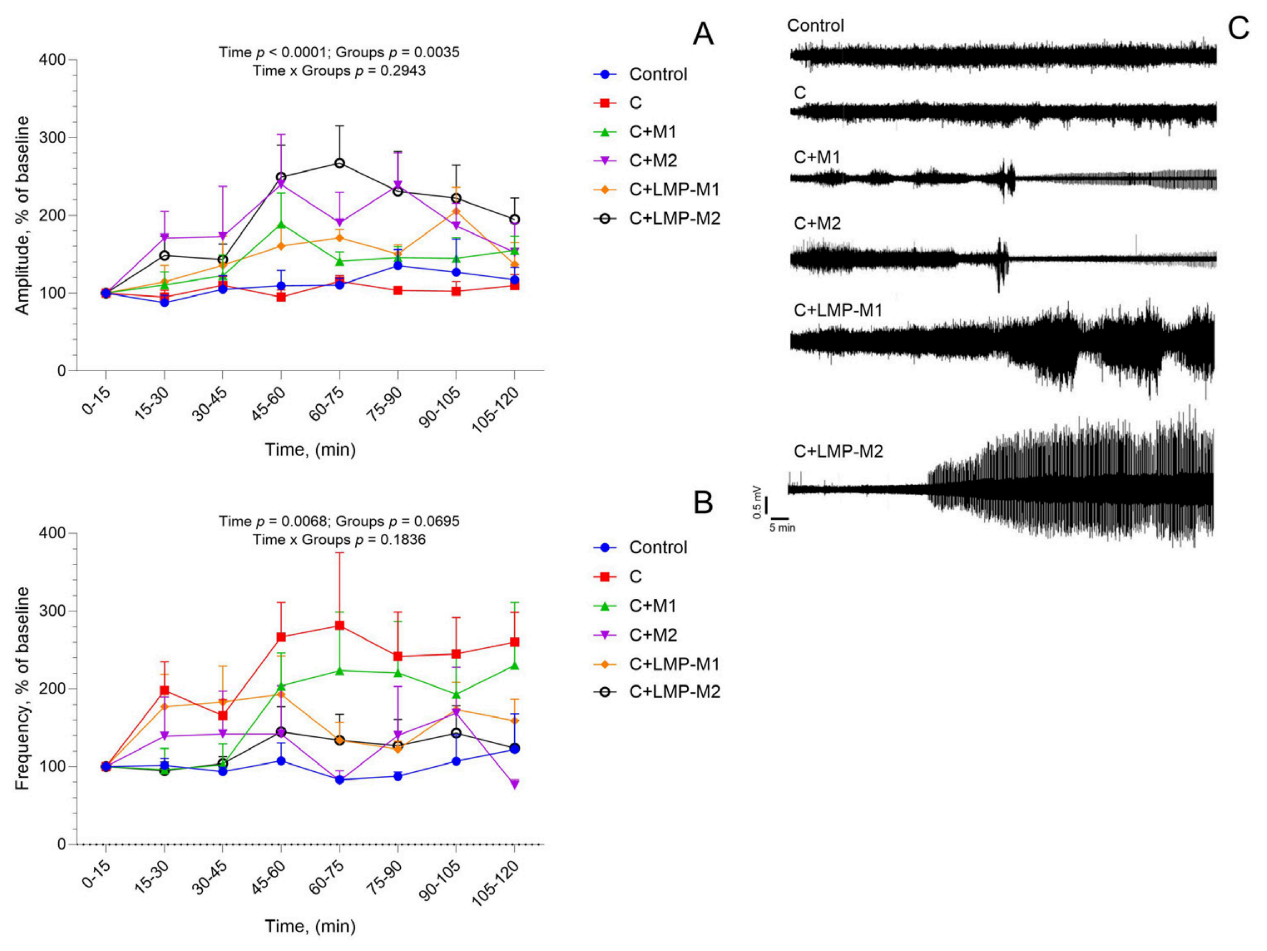
Effect of different forms and doses of caffeine and melatonin on brain electrical activity. Amplitude (**A**) and frequency (**B**) analyses in ECoG recordings (**C**). Data are presented as mean ± SEM (*n* = 7). Control (oral placebo + i.p. saline), C: Caffeine (7.5 mg/kg, i.p.), C+M1: Caffeine+melatonin (10 mg/kg, oral), C+M2: Caffeine+melatonin (20 mg/kg, oral), C+LMP–M1: Caffeine+LMP—melatonin (10 mg/kg, oral), C+LMP–M2: Caffeine+LMP–melatonin (20 mg/kg, oral).

Figure 1A,B summarizes the time-dependent changes in cortical electrical activity following caffeine and melatonin administration under urethane anesthesia. For normalized ECoG amplitude, a significant effect of time (*p* < 0.0001) and an overall effect of treatment group (*p* = 0.0235) were observed, whereas the time × group interaction was not significant (*p* = 0.2943; Figure 1A). Amplitude remained relatively close to baseline in the Control and caffeine-only groups, while the melatonin-treated groups generally showed greater numerical increases. The highest mean amplitude values were observed in the C+LMP-M2 group, reaching approximately 250–260% of baseline during the 45–75 min period. The C+M2 group also displayed transient elevations, with mean values of approximately 200–230% of baseline at several time points. However, the absence of a significant time × group interaction indicates that these temporal patterns should not be interpreted as statistically distinct treatment-specific responses.

Normalized spike frequency showed considerable variability across time and treatment groups (Figure 1B). Neither the main effect of time (*p* = 0.0868) nor the overall group effect (*p* = 0.0695) reached statistical significance, and the time × group interaction was also not significant (*p* = 0.1836). The caffeine group exhibited a numerical increase in spike frequency, reaching approximately 270–280% of baseline during the 45–75 min interval. Although the C+LMP-M2 group generally displayed lower mean values than the caffeine group during this period, the statistical analysis does not support a definitive attenuation of caffeine-induced spike activity by either standard melatonin or LMP-melatonin. Representative ECoG traces from each experimental group are presented in Figure 1C to illustrate the observed waveform characteristics. These traces are qualitative examples and were not used as independent evidence of statistical significance.

### 2.2. Effects of Melatonin Formulations on Pentobarbital-Induced Sedative-Hypnotic Responses

**Figure 2:**
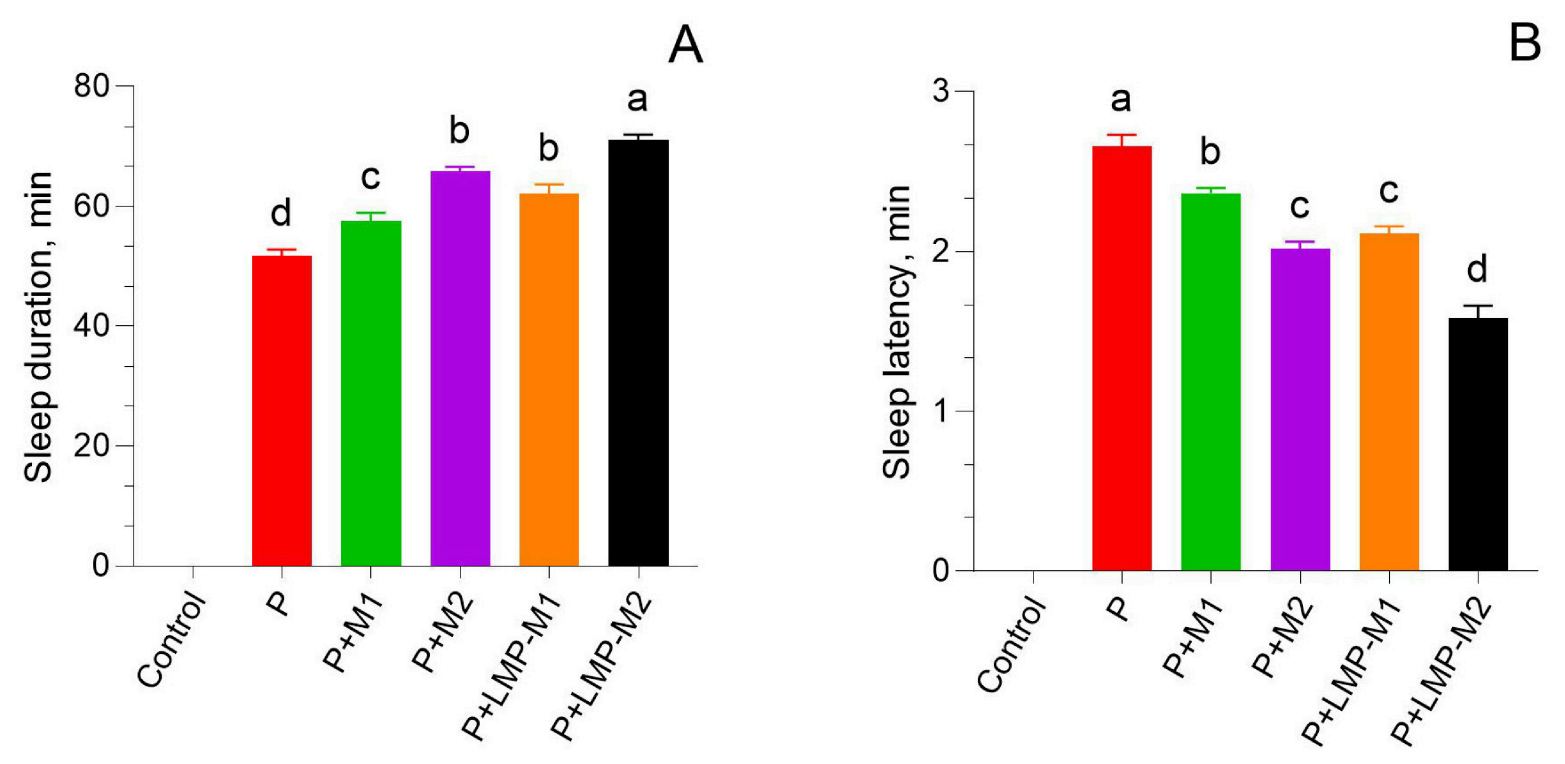
Effects of standard melatonin and LMP-melatonin on sleep duration (**A**) and sleep latency (**B**) in pentobarbital-treated mice. Data are presented as mean ± SEM (*n* = 7). Groups: Control (saline), P (pentobarbital alone, 42 mg/kg, i.p.), P+M1 (pentobarbital+standard melatonin, 10 mg/kg), P+M22 (pentobarbital+standard melatonin, 20 mg/kg), P+LMP–M1 (pentobarbital+LMP–melatonin, 10 mg/kg), and P+LMP–M2 (pentobarbital+LMP–melatonin, 20 mg/kg). Intergroup comparisons were performed using one-way ANOVA followed by Tukey’s post hoc test. Different lowercase letters (a–d) indicate statistically significant differences among groups (*p <* 0.05).

Figure 2 illustrates the effects of standard and LMP-melatonin on pentobarbital-induced sleep. Control animals treated with saline did not enter sleep, whereas administration of pentobarbital (42 mg/kg, i.p.) consistently induced sleep. Melatonin significantly increased the sleep duration in a dose-dependent manner compared to pentobarbital alone (P vs. P+M1, *p* = 0.0095; P vs. P+M22, *p <* 0.0001), with the higher dose being more effective than the lower dose (P+M22 vs. P+M1, *p* = 0.0001). Likewise, treatment with standard melatonin statistically decreased sleep latencies (P vs. P+M1, *p* = 0.0103; P vs. P+M22, *p <* 0.0001), and this effect had a shorter onset with the higher dose of melatonin as compared to the low dose (P+M22 vs. P+M1, *p* = 0.0017). LMP-melatonin had a significantly more robust effect than standard melatonin on sleep duration and sleep latency at equivalent doses. Among the groups, the high-dose LMP-melatonin group (P+LMP-M2) had the longest sleep duration and the shortest sleep latency. P+LMP-M2 significantly improved sleep duration and latency over P+M1 (*p <* 0.0001 for both). Direct comparisons between high-dose formulations indicated that LMP-melatonin was more effective (P+M22 vs. P+LMP-M2; sleep duration *p* = 0.0254; sleep latency *p <* 0.0001). Increasing the dose of LMP-melatonin produced a further increase in both sleep duration and sleep-onset effects (P+LMP-M1 vs. P+LMP-M2; both *p <* 0.0001).

### 2.3. Effects of Standard and LMP-Melatonin on Circulating Neurochemical and Inflammatory Markers in a Caffeine-Induced Insomnia Model

**Figure 3:**
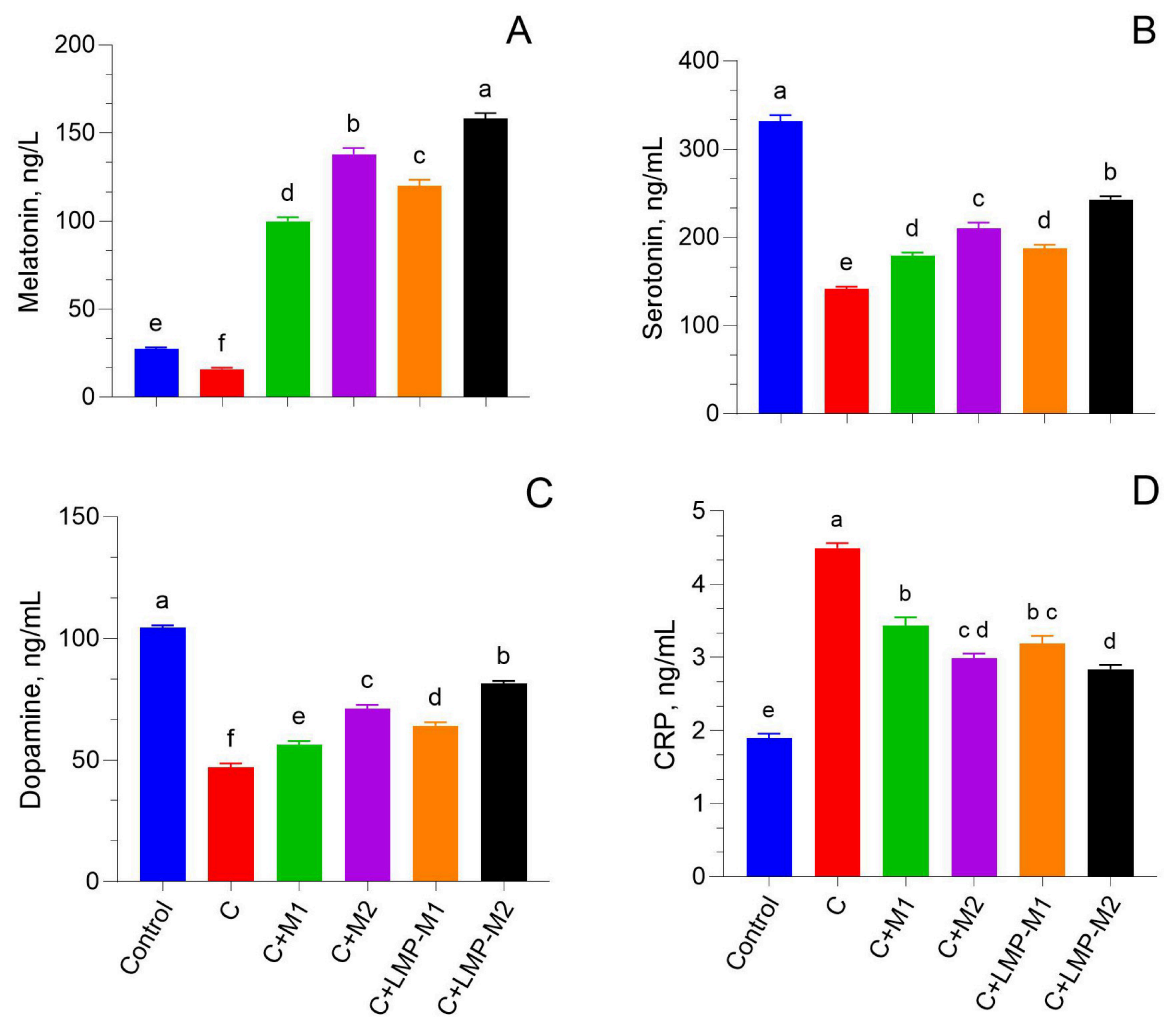
Effects of standard melatonin and LMP-melatonin administered in caffeine-treated mice on serum melatonin (**A**), serotonin (**B**), dopamine (**C**), and C-reactive protein (CRP) (**D**) levels. Data are presented as mean ± SEM (*n* = 7). Groups: Control (oral placebo+i.p. saline), C (caffeine alone, 7.5 mg/kg, i.p.), C+M1 (caffeine+standard melatonin, 10 mg/kg, oral), C+M2 (caffeine+standard melatonin, 20 mg/kg, oral), C+LMP–M1 (caffeine+LMP–melatonin, 10 mg/kg, oral), C+LMP–M2 (caffeine + LMP–melatonin, 20 mg/kg, oral). One-way ANOVA and Tukey post hoc tests were used for intergroup comparisons. Different lowercase letters (a–f) indicate a statistically significant difference between groups (*p <* 0.05).

Caffeine administration substantially disrupted circulating neurochemical profiles, with serum melatonin, serotonin, and dopamine significantly lower in the C group compared with controls; CRP was higher (Control vs. C, all *p <* 0.0001; Figure 3A–D). Standard melatonin dose-dependently restored serum melatonin (C vs. C+M1, C+M2; both *p <* 0.0001), but LMP-melatonin led to significantly greater peak levels at similar doses, with the maximal level observed in the high-dose LMP group (C+M2 vs. C+LMP-M2; *p <* 0.0001). The same pattern was found for serotonin and dopamine. Melatonin treatment (LMP-melatonin being the most effective and particularly at the high dose) significantly reversed caffeine-induced depletion of both neurotransmitters (serotonin: Control vs. C+LMP-M2, *p <* 0.0001; C+M2 vs. C+LMP-M2, *p* = 0.0004; dopamine: Control vs. C, *p <* 0.0001; C+LMP-M1 vs. C+LMP-M2, *p <* 0.00001). Caffeine also markedly increased serum CRP concentration, indicating an enhanced systemic inflammatory response. Both standard and LMP-melatonin reduced CRP relative to the caffeine-only group, with the lowest concentration observed in the C+LMP-M2 group (Figure 3D).

### 2.4. Effects of Standard and LMP-Melatonin on Oxidative Stress Parameters

**Figure 4:**
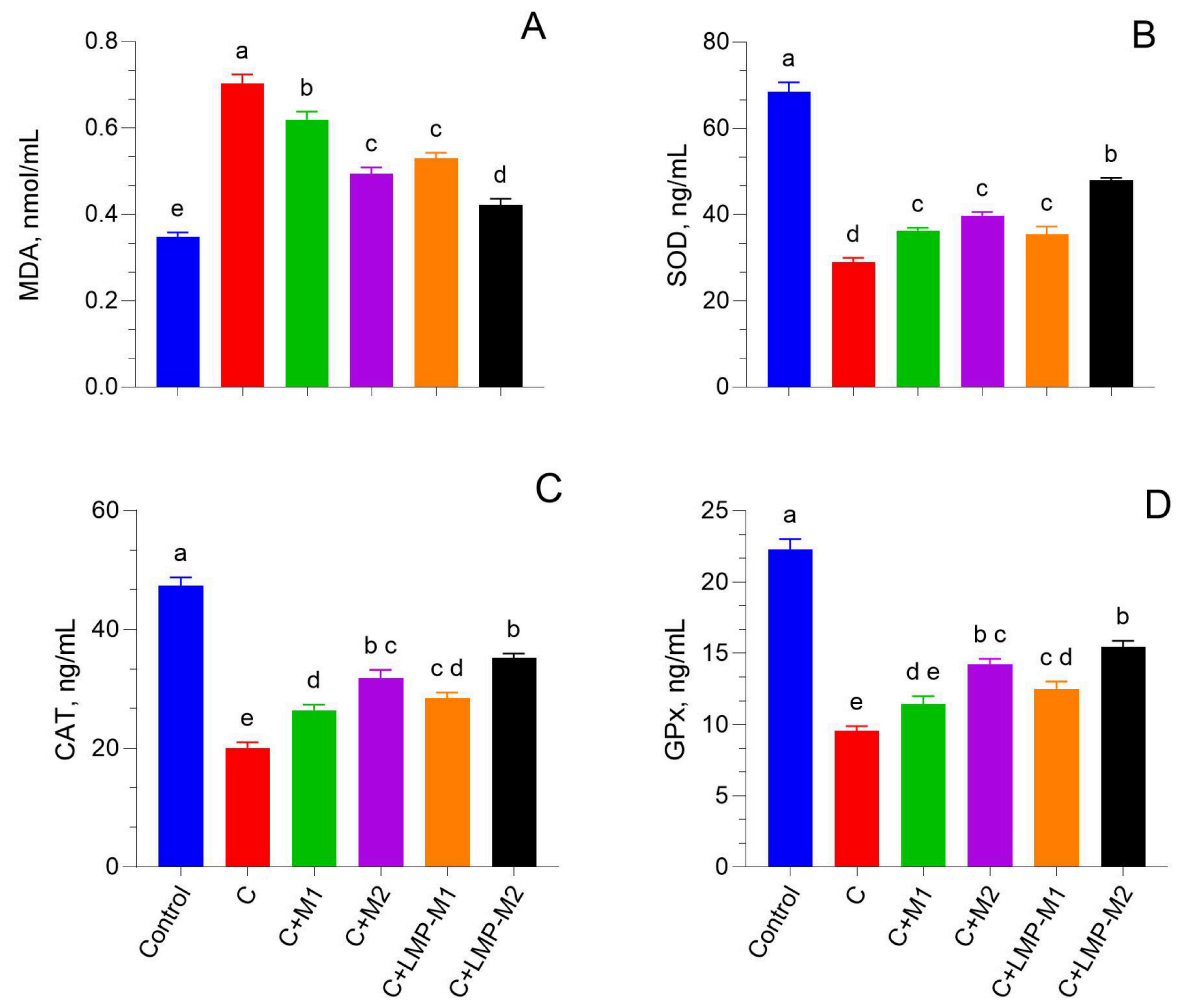
Effects of standard melatonin and LMP-melatonin on serum oxidative stress parameters in a caffeine-induced insomnia model. Malondialdehyde (MDA, nmol/mL) (**A**); Superoxide dismutase (SOD, ng/mL) (**B**); Catalase (CAT, ng/mL) (**C**); Glutathione peroxidase (GPx, ng/mL) (**D**). Data are presented as mean ± SEM (*n* = 7). Groups: Control (oral placebo+i.p. saline), C (caffeine alone, 7.5 mg/kg, i.p.), C+M1 (caffeine+standard melatonin, 10 mg/kg, oral), C+M2 (caffeine+standard melatonin, 20 mg/kg, oral), C+LMP–M1 (caffeine+LMP–melatonin, 10 mg/kg, oral), C+LMP–M2 (caffeine+LMP–melatonin, 20 mg/kg, oral). One-way ANOVA and Tukey post hoc tests were used for intergroup comparisons. Different lowercase letters (a–e) indicate a statistically significant difference between groups (*p <* 0.05).

Caffeine treatment severely disturbed redox homeostasis, as evidenced by elevated lipid peroxidation and decreased antioxidant enzyme concentrations relative to controls (Control vs. C, all *p <* 0.0001; Figure 4A–D). Following caffeine administration, serum MDA levels were significantly increased, and both standard and LMP-melatonin could ameliorate this elevation in a dose-dependent manner. Melatonin in standard formulation decreased serum MDA levels compared to caffeine only (C vs. C+M1, *p* = 0.0083; C vs. C+M2, *p <* 0.0001), whereas LMP-melatonin provided further suppression; the lowest observed MDA levels were seen with high-dose LMP (C vs. C+LMP-M2, *p <* 0.0001; C+LMP-M1 vs. C+LMP-M2, *p* = 0.0003). Caffeine also significantly decreased serum SOD, CAT, and GPx concentrations, impairing antioxidant defense (Control vs. C; *p <* 0.0001 for all). While both melatonin treatments partially restored enzyme concentrations and levels, LMP-melatonin treatment produced significantly greater increases over the standard formulation (e.g., C+M1 vs C+LMP-M2, all with *p* < 0.0001), and within LMP-treated groups there was a clear dose-dependent effect.

### 2.5. Effects of Standard and LMP-Melatonin on GABAergic and Serotonergic Receptor Expression

**Figure 5:**
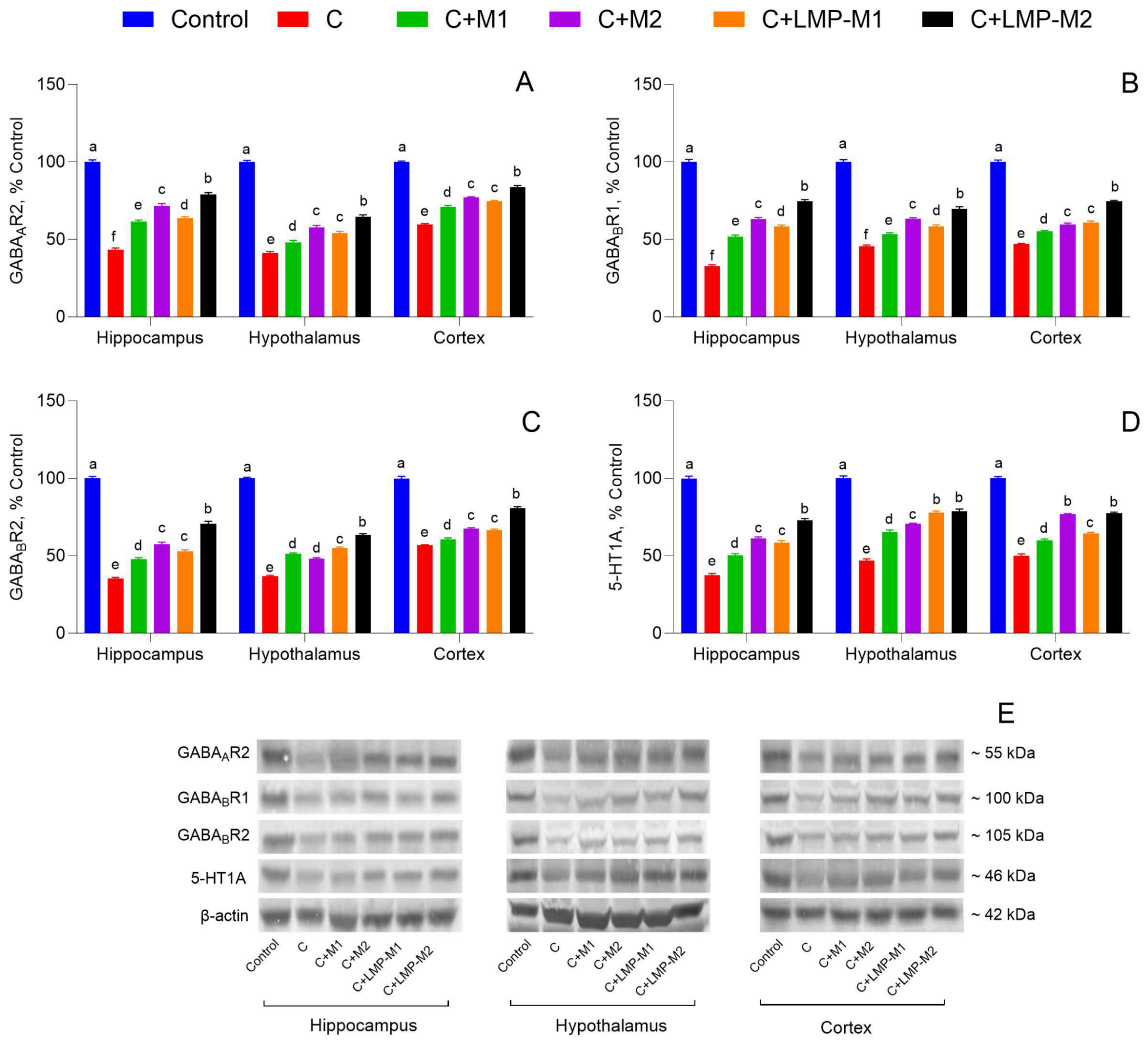
Effects of standard melatonin and LMP-melatonin on GABA_A_R2 (**A**), GABA_B_R1 (**B**), GABA_B_R2 (**C**), and 5-HT1A (**D**) receptor levels in a caffeine-induced insomnia model. Data are expressed in the hippocampus, hypothalamus, and cortex regions. Data are presented as mean ± SEM (*n* = 7). Groups: Control (oral placebo+i.p. saline), C (caffeine alone, 7.5 mg/kg, i.p.), C+M1 (caffeine+standard melatonin, 10 mg/kg, oral), C+M2 (caffeine+standard melatonin, 20 mg/kg, oral), C+LMP–M1 (caffeine+LMP–melatonin, 10 mg/kg, oral), C+LMP–M2 (caffeine+LMP–melatonin, 20 mg/kg, oral). For each target, one-way ANOVA followed by Tukey’s post hoc test was performed separately within each brain region. Within the hippocampus, hypothalamus, and cortex, bars that do not share a common lowercase letter differ significantly (*p* < 0.05). Lettering was assigned independently for each brain region and does not indicate comparisons between regions. Western blot analysis was performed with the included β-actin to ensure equal protein loading. All bands are shown in (**E**).

**Figure 6:**
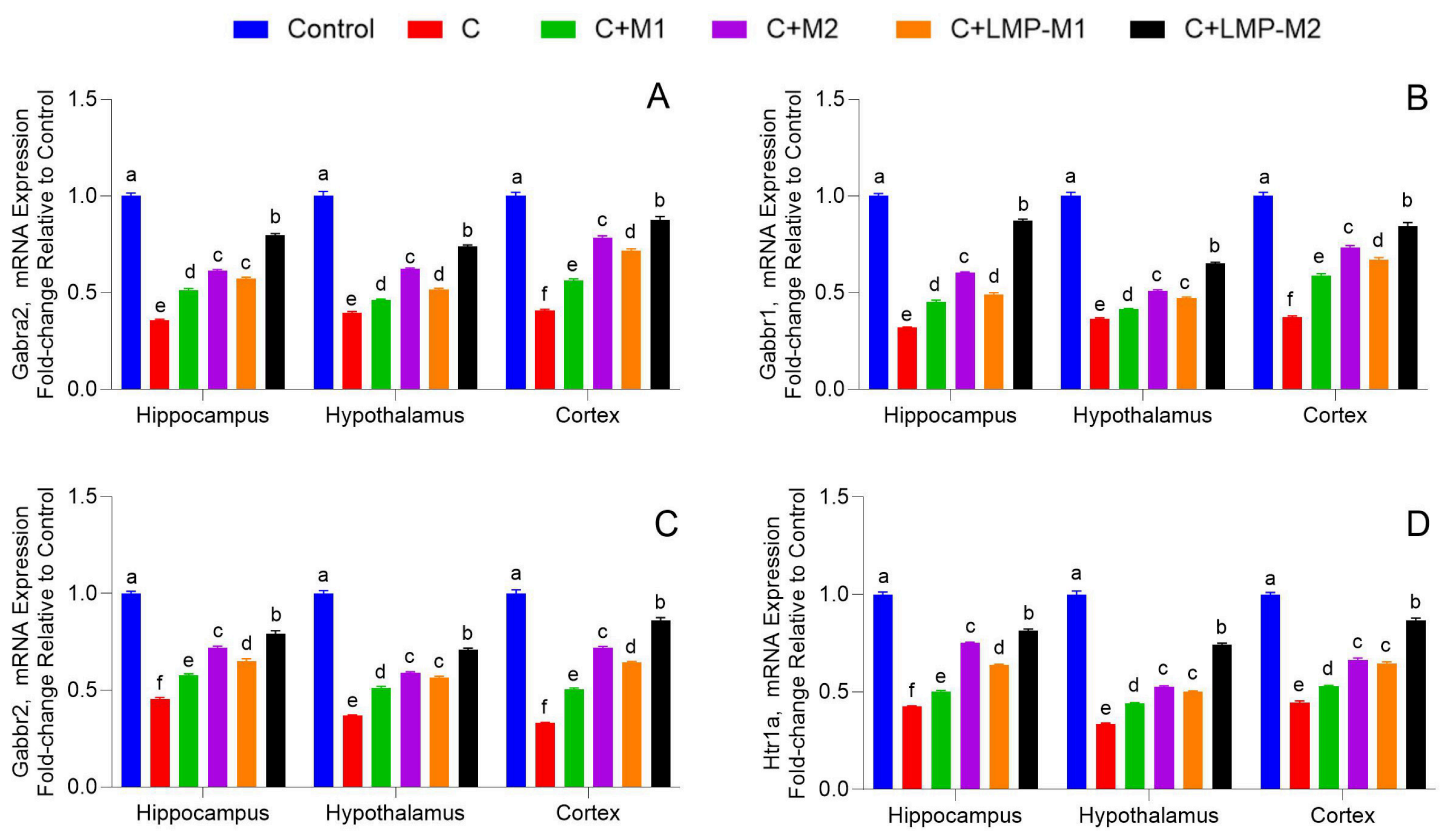
Effects of standard melatonin and LMP-melatonin on GABAergic and serotonergic receptor mRNA expression in a caffeine-induced insomnia model. Relative mRNA expression levels of Gabra2 (**A**), Gabbr1 (**B**), Gabbr2 (**C**), and Htr1a (**D**) were quantified in the hippocampus, hypothalamus, and cortex of mice exposed to the caffeine-evoked arousal model. Gene expression was normalized to the internal reference gene and expressed as fold change relative to the control group. Data are presented as mean ± SEM (*n* = 7). Groups: Control (oral placebo+intraperitoneal saline), C (caffeine alone, 7.5 mg/kg, i.p.), C+M1 (caffeine+standard melatonin, 10 mg/kg, oral), C+M2 (caffeine+standard melatonin, 20 mg/kg, oral), C+LMP–M1 (caffeine+LMP–melatonin, 10 mg/kg, oral), and C+LMP–M2 (caffeine+LMP–melatonin, 20 mg/kg, oral). For each target, one-way ANOVA followed by Tukey’s post hoc test was performed separately within each brain region. Within the hippocampus, hypothalamus, and cortex, bars that do not share a common lowercase letter differ significantly (*p* < 0.05). Lettering was assigned independently for each brain region and does not indicate comparisons between regions.

There were significant reductions in GABAergic and serotonergic signaling throughout the hippocampus, hypothalamus, and cortex (in all regions: *p <* 0.0001) following caffeine exposure, as evidenced by diminished expression of GABA_A_R2, GABA_B_R1, GABA_B_R2, and 5–HT11A protein (Figure 5 and Figure S1) and mRNA levels (Figure 6) compared to controls. Melatonin increased receptor expression at the transcriptional and protein levels; however, levels did not return to control values in some brain regions tested (but were dose-dependent). The C+LMP-M2 group (high-dose LMP-melatonin) had the highest protein abundance and mRNA expression in all regions, significantly higher than caffeine and standard melatonin–treated groups (*p <* 0.01 to <0.0001). Intermediate effects were observed for low-dose LMP-melatonin and high-dose melatonin standard, whereas modest efficacy resulted from low-dose melatonin standard.

### 2.6. Effects of Standard and LMP-Melatonin on Glutamatergic Receptor Subunit Expression

**Figure 7:**
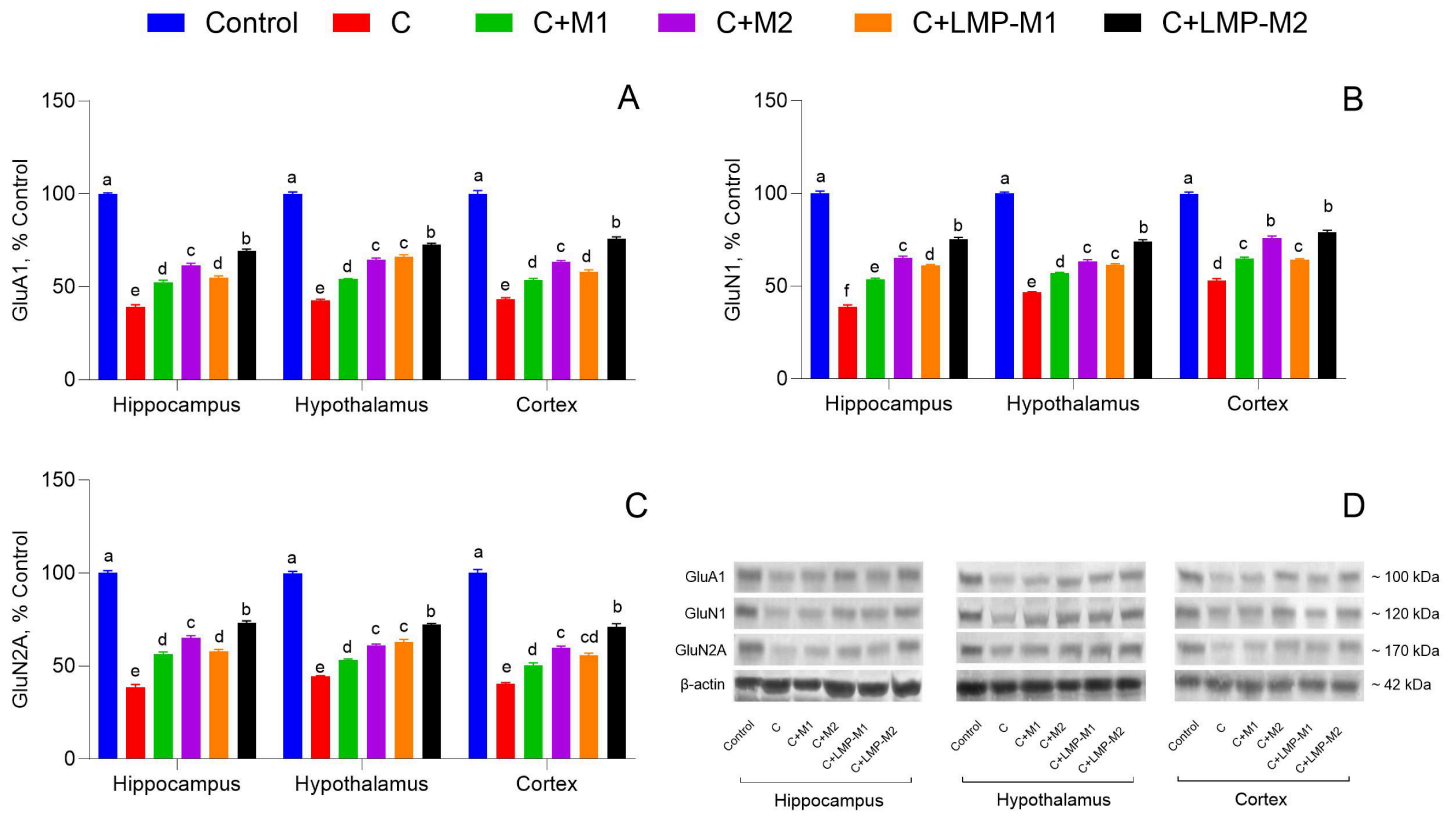
Effects of standard melatonin and LMP-melatonin on Glu receptor subunits GluA1 (**A**), GluN1 (**B**), and GluN2A (**C**) levels in a caffeine-induced insomnia model. Receptor expression is shown as % control in the hippocampus, hypothalamus, and cortex. Data are presented as mean ± SEM (*n* = 7). Groups: Control (oral placebo + i.p. saline), C (caffeine alone, 7.5 mg/kg, i.p.), C+M1 (caffeine + standard melatonin, 10 mg/kg, oral), C+M2 (caffeine+standard melatonin, 20 mg/kg, oral), C+LMP–M1 (caffeine+LMP–melatonin, 10 mg/kg, oral), C+LMP–M2 (caffeine+LMP–melatonin, 20 mg/kg, oral). For each target, one-way ANOVA followed by Tukey’s post hoc test was performed separately within each brain region. Within the hippocampus, hypothalamus, and cortex, bars that do not share a common lowercase letter differ significantly (*p* < 0.05). Lettering was assigned independently for each brain region and does not indicate comparisons between regions. Western blot analysis was performed with the included β-actin to ensure equal protein loading. All bands are shown in (**D**).

**Figure 8:**
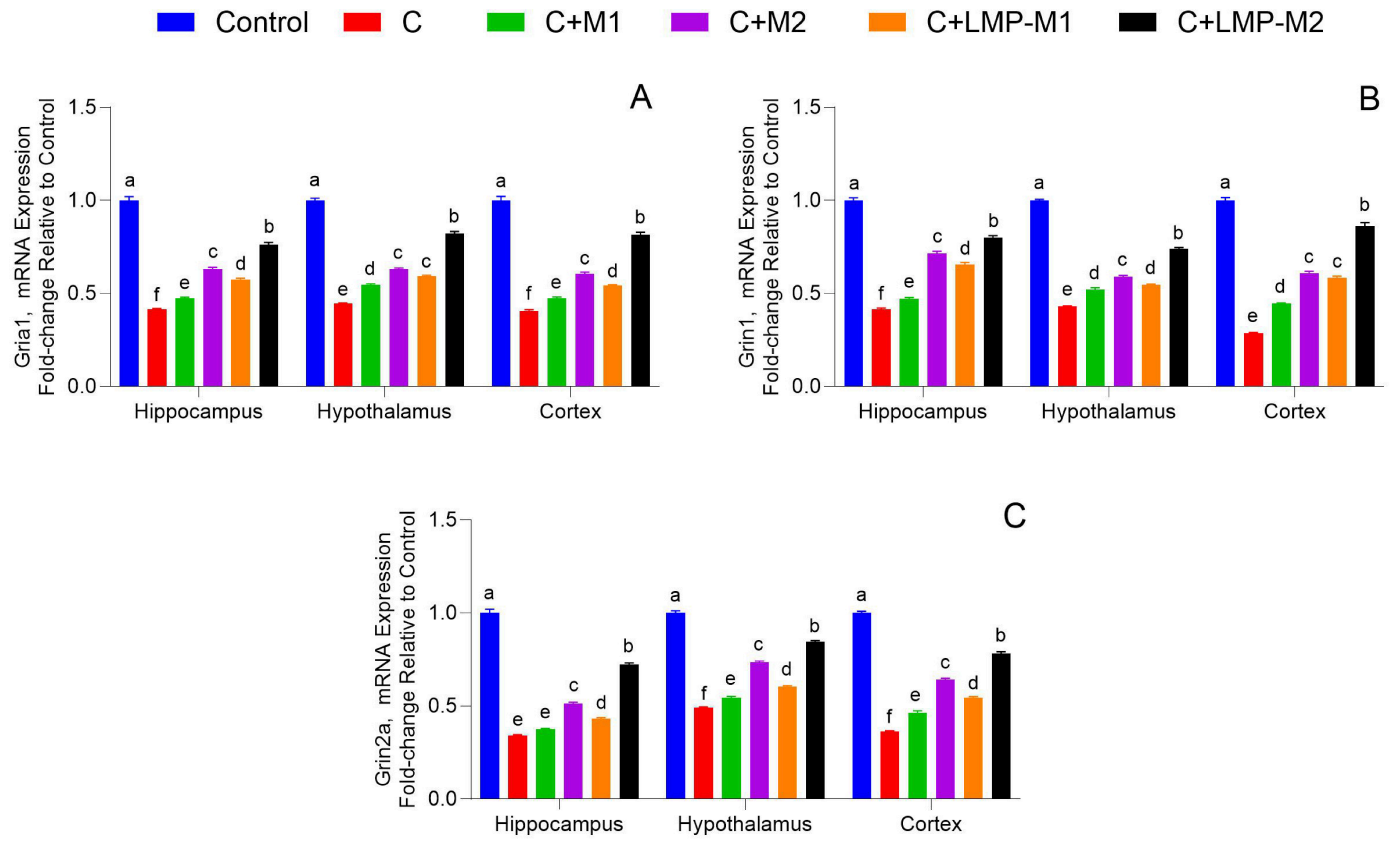
Effects of standard melatonin and LMP-melatonin on glutamatergic receptor subunit mRNA expression in a caffeine-induced insomnia model. Relative mRNA expression levels of GluA1 (AMPA receptor subunit 1) (**A**), GluN1 (NMDA receptor subunit 1) (**B**), and GluN2A (NMDA receptor subunit 2A) (**C**) were measured in the hippocampus, hypothalamus, and cortex of mice subjected to caffeine-evoked arousal. Gene expression levels were normalized to the internal reference gene and expressed as fold change relative to the control group. Data are presented as mean ± SEM (*n* = 7). Groups: Control (oral placebo+intraperitoneal saline), C (caffeine alone, 7.5 mg/kg, i.p.), C+M1 (caffeine+standard melatonin, 10 mg/kg, oral), C+M2 (caffeine+standard melatonin, 20 mg/kg, oral), C+LMP–M1 (caffeine+LMP–melatonin, 10 mg/kg, oral), and C+LMP–M2 (caffeine+LMP–melatonin, 20 mg/kg, oral). For each target, one-way ANOVA followed by Tukey’s post hoc test was performed separately within each brain region. Within the hippocampus, hypothalamus, and cortex, bars that do not share a common lowercase letter differ significantly (*p* < 0.05). Lettering was assigned independently for each brain region and does not indicate comparisons between regions.

Administration of caffeine significantly reduced expression of glutamatergic receptor subunits across the hippocampus, hypothalamus and cortex, as shown by significant reductions in GluA1 (*p <* 0.0001), GluN1 (*p <* 0.0001), and GluN2A (*p <* 0.001) protein levels compared to control (Figure 7) and mRNA levels compared to controls (all regions *p* < 0.0001; Figure 8 and Figure S2). Standard melatonin partially corrected caffeine-induced deficits in glutamatergic receptor subunit expression in a dose-dependent manner, although restoration remained incomplete in several brain regions. Among the LMP-melatonin–treated groups, the high-dose LMP-melatonin group (C+LMP-M2) exhibited the highest protein abundance and mRNA expression levels for GluA1, GluN1, and GluN2A across the hippocampus, hypothalamus, and cortex. These values were significantly greater than those observed in the caffeine-only and standard melatonin-treated groups (*p <* 0.001 to *p <* 0.0001) and approached control levels, particularly in the hippocampus and cortex. Low-dose LMP-melatonin and high-dose standard melatonin produced intermediate improvements, whereas low-dose standard melatonin showed comparatively modest effects.

### 2.7. Effects of Standard and LMP-Melatonin on Central Inflammatory Markers

**Figure 9:**
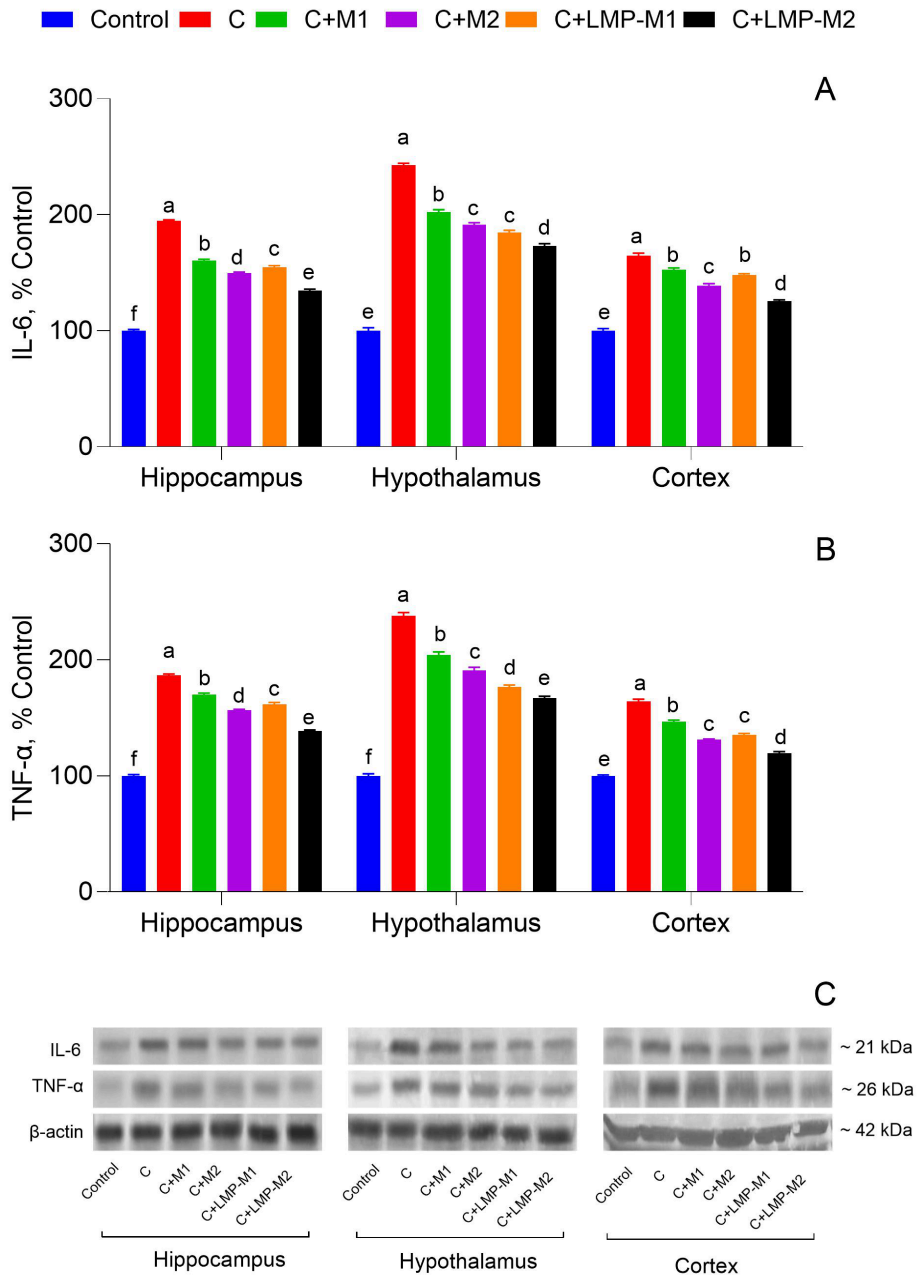
Effect of standard melatonin and LMP-melatonin on inflammatory markers IL-6 (**A**) and TNF-α (**B**) levels in a caffeine-induced insomnia model. Cytokine levels are expressed as % control in the hippocampus, hypothalamus, and cortex. Data are presented as mean ± SEM (*n* = 7). Groups: Control (oral placebo+i.p. saline), C (caffeine alone, 7.5 mg/kg, i.p.), C+M1 (caffeine+standard melatonin, 10 mg/kg, oral), C+M2 (caffeine+standard melatonin, 20 mg/kg, oral), C+LMP–M1 (caffeine+LMP–melatonin, 10 mg/kg, oral), C+LMP–M2 (caffeine+LMP–melatonin, 20 mg/kg, oral). For each target, one-way ANOVA followed by Tukey’s post hoc test was performed separately within each brain region. Within the hippocampus, hypothalamus, and cortex, bars that do not share a common lowercase letter differ significantly (*p* < 0.05). Lettering was assigned independently for each brain region and does not indicate comparisons between regions. Western blot analysis was performed with the included β-actin to ensure equal protein loading. All bands are shown in (**C**).

**Figure 10:**
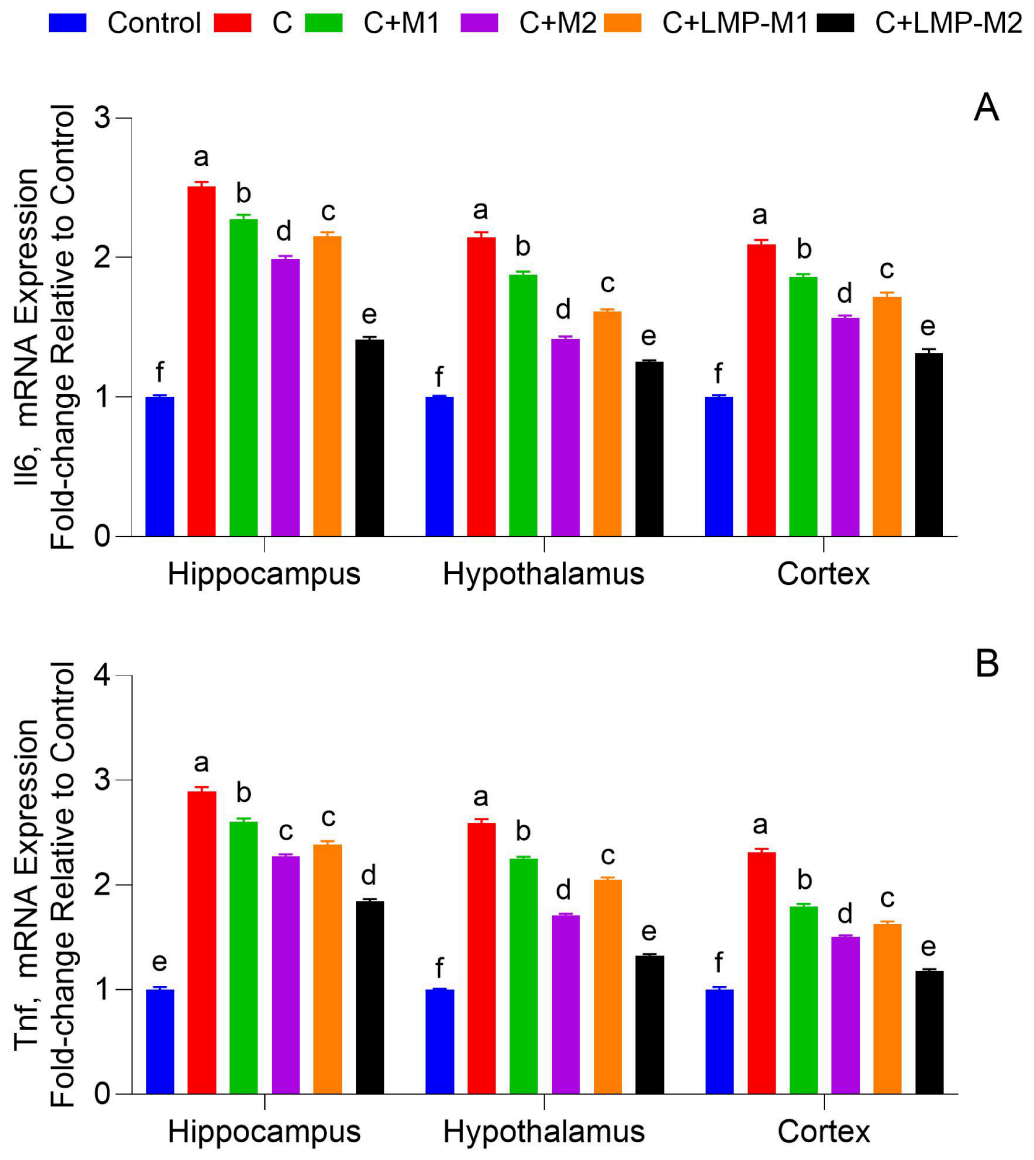
Effects of standard melatonin and LMP-melatonin on pro-inflammatory cytokine mRNA expression in a caffeine-induced insomnia model. Relative mRNA expression levels of Il6 (**A**) and Tnf (**B**) were quantified in the hippocampus, hypothalamus, and cortex of mice subjected to caffeine-evoked arousal. Gene expression levels were normalized to the internal reference gene and expressed as fold change relative to the control group. Data are presented as mean ± SEM (*n* = 7). Groups: Control (oral placebo+intraperitoneal saline), C (caffeine alone, 7.5 mg/kg, i.p.), C+M1 (caffeine+standard melatonin, 10 mg/kg, oral), C+M2 (caffeine+standard melatonin, 20 mg/kg, oral), C+LMP–M1 (caffeine+LMP–melatonin, 10 mg/kg, oral), and C+LMP–M2 (caffeine+LMP–melatonin, 20 mg/kg, oral). For each target, one-way ANOVA followed by Tukey’s post hoc test was performed separately within each brain region. Within the hippocampus, hypothalamus, and cortex, bars that do not share a common lowercase letter differ significantly (*p* < 0.05). Lettering was assigned independently for each brain region and does not indicate comparisons between regions.

Caffeine exposure induced a robust neuroinflammatory response across the hippocampus, hypothalamus, and cortex, as reflected by significant increases in IL-6 and TNF-α protein levels (Figure 9 and Figure S3) and mRNA expression (Figure 10) compared with control (all regions, *p <* 0.0001). Standard melatonin attenuated caffeine-induced cytokine elevations in a dose-dependent manner but did not fully normalize levels across regions. LMP-melatonin produced a significantly stronger and more consistent suppression of IL-6 and TNF-α expression. The high-dose LMP-melatonin group (C+LMP-M2) exhibited the lowest cytokine levels among caffeine-treated animals, with values significantly lower than those of both the caffeine and standard melatonin groups (*p <* 0.05) and approaching control levels, particularly in the hypothalamus. Similar patterns were observed at the transcriptional level, where LMP-melatonin, especially at the higher dose, most effectively suppressed caffeine-induced IL-6 and TNF-α gene expression (C+LMP-M2 vs. C and standard melatonin groups, *p <* 0.05).

## 3. Discussion

The present study compared standard and LMP-melatonin in two complementary acute pharmacological paradigms. The ECoG experiment evaluated caffeine-evoked changes in cortical electrical activity under urethane anesthesia, whereas the pentobarbital experiment assessed sedative-hypnotic responsiveness using loss of the righting reflex. Caffeine produced a time-dependent increase in spike frequency and altered ECoG amplitude, accompanied by unfavorable changes in circulating neurochemical and redox markers and in molecular outcomes related to neurotransmitter receptors and inflammation. Both melatonin formulations attenuated several of these alterations in a dose-dependent manner, with the strongest effects generally observed in the high-dose LMP-melatonin group. Compared with conventional melatonin, the LMP formulation produced greater modulation of spike frequency, stronger enhancement of pentobarbital-induced sedative-hypnotic responses, and more favorable changes in circulating biochemical markers, neurotransmitter-receptor expression, and IL-6 and TNF-α-related outcomes.

The marked increase in ECoG amplitude observed with high-dose LMP-melatonin should nevertheless be interpreted cautiously. Amplitude alone does not provide sufficient evidence of cortical synchronization, slow-wave activity, or physiological sleep promotion. Because recordings were obtained under urethane anaesthesia and spectral power, coherence, and simultaneous EEG/EMG-based sleep staging were not assessed, the electrophysiological findings represent treatment-associated changes in cortical electrical activity rather than normalization of natural sleep-related activity. Furthermore, the observed formulation-dependent differences do not establish enhanced bioavailability, increased brain exposure, MT1/MT2 activation, or engagement of specific intracellular signaling pathways, as these mechanisms were not directly evaluated. Future studies incorporating pharmacokinetic profiling and EEG/EMG recordings in freely moving animals are required to determine the relationships between these effects and natural wakefulness, NREM sleep, and REM sleep.

Caffeine administration produced time-dependent changes in cortical electrical activity, including increased spike frequency and altered amplitude. This electrophysiological profile is consistent with adenosine A1/A2A receptor antagonism, a mechanism widely recognized to reduce sleep pressure and promote arousal-related neural activity. Previous human and animal studies have reported that caffeine can reduce slow-wave EEG activity and alter the spectral characteristics of cortical activity during sleep [26,27]. However, because spectral power was not assessed in the present study, our findings are limited to changes in ECoG amplitude and spike frequency under urethane anesthesia. Melatonin attenuated the caffeine-induced increase in spike frequency in a dose-dependent manner, with the strongest reduction observed in the high-dose LMP-melatonin group. However, because spectral power was not assessed, no conclusions could be drawn about synchronized oscillatory activity. Previous studies have shown that melatonin can modulate sleep-related EEG activity and reduce neuronal excitability through effects on voltage-gated ion channels [28,29]; however, spectral changes were not evaluated in the present study. Compared with conventional melatonin, the LMP formulation produced a more sustained modulation of caffeine-evoked ECoG amplitude and spike frequency. The electrophysiological findings were corroborated behaviorally in the pentobarbital assay, in which melatonin enhanced hypnotic responses in a dose-dependent manner. LMP-melatonin produced a significantly shortened sleep latency and extended total sleep duration compared to standard melatonin. Since pentobarbital-induced sleep is a measure of GABA_A receptor–mediated inhibitory facilitation, this reinforced effect upon LMP delivery likely reflects increased potentiation of the inhibitory circuitry [30,31]. At least in methodological and mechanistic terms, pharmacokinetic analyses consistently show that sustained systemic exposure increases the magnitude of melatonin’s hypnotic effect and prolongs its duration [32,33], providing a mechanistic basis for the much-improved behavioral results evident here. The two experimental paradigms should be interpreted within their methodological scope. ECoG recordings were obtained under urethane anaesthesia after surgical exposure of the cortex and therefore reflect caffeine-evoked changes in cortical electrical activity in an anaesthetised preparation rather than insomnia or physiological sleep in freely moving mice. Although urethane can produce alternating sleep-like brain states, these states are not equivalent to natural wakefulness, NREM sleep, or REM sleep. Accordingly, the observed effects of melatonin are interpreted as modulation of caffeine-evoked cortical activity and excitability. Similarly, the pentobarbital experiment measured the latency to and duration of loss of the righting reflex, which are established indicators of sedative-hypnotic activity but do not provide information on physiological sleep architecture or sleep quality. Therefore, the stronger response observed with LMP-melatonin indicates greater potentiation of the pentobarbital-induced hypnotic response rather than restoration of natural sleep.

Caffeine administration was associated with lower circulating concentrations of melatonin, serotonin, and dopamine, and with higher CRP levels. Melatonin treatment attenuated these changes, with generally greater effects observed for the LMP formulation. However, circulating serotonin and dopamine concentrations do not directly represent neurotransmitter release or activity within the central nervous system and should therefore be interpreted as peripheral biochemical indicators rather than direct measures of central monoaminergic neurotransmission. Adenosine receptor antagonism is known to alter monoaminergic neurotransmission and promote arousal-related neural activity, thereby disrupting sleep–wake regulation [24,25]. Sleep disruption itself promotes low-grade systemic inflammation [34]. The reduction in CRP is consistent with the previously reported anti-inflammatory properties of melatonin [15,35,36]. Although earlier studies have implicated NF-κB and inflammasome pathways in these effects [16,37,38], neither pathway was directly evaluated in the present study.

Caffeine exposure was further associated with marked oxidative stress, evidenced by elevated lipid peroxidation and suppression of endogenous antioxidant enzymes, a profile consistent with both clinical insomnia and experimental sleep restriction models [39,40]. Caffeine increased lipid peroxidation and reduced endogenous antioxidant-enzyme concentrations, whereas both melatonin formulations lowered MDA and increased SOD, CAT, and GPx concentrations. These findings indicate an improvement in the measured redox profile. Previous studies have linked melatonin’s antioxidant effects to direct radical scavenging and to the regulation of Nrf2/ARE-dependent pathways [41,42]. However, Nrf2 expression, nuclear translocation, DNA binding, and downstream ARE-regulated proteins were not measured; therefore, activation of this pathway cannot be concluded from the present results.

Caffeine exposure was associated with reduced expression of GABAergic, serotonergic, and glutamatergic receptor-related targets in the cortex, hippocampus, and hypothalamus. Decreased expression of GABA receptor subunits may indicate an altered molecular capacity for inhibitory neurotransmission, whereas changes in 5-HT 1A expression may be relevant to mechanisms involved in sleep initiation and maintenance [43,44,45]. Previous studies have shown that adenosine-receptor antagonism can modify monoaminergic and GABAergic signaling and increase cortical and hippocampal excitability [46,47]. In parallel, reductions in GluA1 and GluN1/GluN2A suggest alterations in molecular systems associated with AMPA- and NMDA-receptors. Given the established roles of these receptors in synaptic plasticity, sleep-dependent synaptic renormalization, and memory consolidation [48,49], their reduced expression may be consistent with impaired synaptic homeostasis during sustained arousal. However, receptor-expression measurements alone cannot establish functional reductions in synaptic inhibition, alterations in receptor-mediated currents, or disruption of inhibitory–excitatory network balance.

Both melatonin formulations attenuated these caffeine-associated changes, with generally greater effects observed for LMP-melatonin. Previous studies have linked melatonin’s neuromodulatory actions to MT1/MT2 receptor signaling, facilitation of inhibitory synaptic transmission, and regulation of serotonergic neuronal activity [50,51]. Similarly, the improvement in GluA1 and GluN1/GluN2A expression is consistent with earlier evidence suggesting that melatonin may support synaptic integrity and plasticity under stress conditions [52,53]. Nevertheless, MT1/MT2 receptor expression or activation, downstream intracellular signaling, receptor-mediated currents, and functional synaptic activity were not directly evaluated in the present study. The greater effects observed with LMP-melatonin may also be compatible with formulation-dependent differences in release, absorption, or duration of exposure previously described [54,55], as well as reported interactions between melatonergic and serotonergic receptor systems [56]. However, plasma concentration–time profiles, pharmacokinetic parameters, brain melatonin concentrations, and receptor cross-talk were not measured. Therefore, enhanced bioavailability, sustained central exposure, MT1/MT2-dependent signaling, and restoration of inhibitory–excitatory balance should be considered literature-supported mechanistic hypotheses rather than mechanisms demonstrated by the present findings.

Caffeine-associated alterations in receptor expression were accompanied by increased IL-6 and TNF-α expression across the cortex, hippocampus, and hypothalamus. Sleep disruption and circadian dysregulation have previously been linked to enhanced inflammatory signaling within the central nervous system, including increased cytokine production and impaired neuronal function [12,34]. Both melatonin formulations attenuated the caffeine-associated increases in IL-6 and TNF-α, with generally greater reductions observed with LMP-melatonin, particularly in the hypothalamus, an important integrative region for endocrine, immune, and sleep–wake regulation. Previous studies have implicated NF-κB, TLR4/MyD88, inflammasome-related signaling, and microglial activation in melatonin’s anti-inflammatory actions [16,37,57]. However, these pathways, microglial responses, and central melatonin exposure were not directly assessed in the present study. The findings should therefore be interpreted as evidence of formulation-dependent changes in the measured cytokine outcomes rather than direct demonstration of NF-κB, TLR4/MyD88, or inflammasome inhibition or suppression of microglial activation. Moreover, because neuronal injury, apoptosis, histopathological alterations, and cell survival were not evaluated, the present results do not provide direct evidence of neuroprotection. Instead, the measured biochemical and molecular outcomes support neuromodulatory, antioxidant, and anti-inflammatory effects of melatonin, which were generally more pronounced with the LMP formulation.

Although the present findings were obtained in male BALB/c mice, the fundamental mechanisms involved in sleep regulation, including melatonin signaling, neurotransmitter balance, oxidative stress, and inflammatory pathways, are largely conserved across mammalian species. Therefore, the observed effects of LMP-melatonin may have broader relevance beyond this experimental model. However, differences in physiology, metabolism, and sleep architecture between rodents and humans should be considered when interpreting the results. Further studies in additional animal models and well-designed clinical trials are required to determine the translational potential and clinical relevance of LMP-melatonin for the management of sleep disturbances in humans.

Several limitations should be acknowledged. First, the acute caffeine and pentobarbital paradigms do not reproduce the chronic and multifactorial nature of clinical insomnia. ECoG recordings were obtained under urethane anaesthesia following craniotomy rather than in freely moving animals, and both procedures may alter cortical electrical activity. Although ECoG amplitude and spike frequency were evaluated, spectral power, coherence, simultaneous EMG activity, and formal sleep-stage classification were not assessed. Consequently, the observed changes cannot establish cortical synchronization, physiological wakefulness, NREM or REM sleep, sleep architecture, or sleep quality. In particular, the marked increase in ECoG amplitude observed in the high-dose LMP-melatonin group should be interpreted only as a treatment-associated alteration in cortical electrical activity. Similarly, pentobarbital-induced loss of the righting reflex represents a sedative-hypnotic response rather than natural sleep. In addition, BALB/c mice are generally regarded as a melatonin-deficient or very low melatonin-producing strain, although sensitive analytical methods have detected low and transient melatonin rhythms [19,20,21,22]. Because serum samples were collected during the light phase and melatonin was measured using a direct commercial ELISA, measurable control values may have been influenced by assay sensitivity at low concentrations, matrix effects, or cross-reactivity. Serum melatonin results should therefore be interpreted primarily as relative treatment-associated differences rather than definitive estimates of endogenous pineal secretion. Confirmation using an extraction-based assay or LC–MS/MS would be valuable. Only male mice were included, limiting assessment of sex-dependent responses. The absence of an empty LMP-matrix control also prevents exclusion of effects attributable to the lipid carrier itself. Pharmacokinetic profiles and brain melatonin concentrations were not determined; therefore, enhanced systemic bioavailability or sustained central exposure cannot be confirmed. Circulating serotonin and dopamine concentrations should be considered peripheral neurochemical measures and may not reflect central monoaminergic neurotransmission. In addition, the 12 h pretest fasting period may have influenced metabolic, neuroendocrine, or pharmacological responsiveness, including sensitivity to pentobarbital. Finally, neuronal injury, apoptosis, histopathological alterations, and cell survival were not directly evaluated; thus, the findings should not be interpreted as direct evidence of neuroprotection. Future studies should include both sexes, an empty LMP-matrix control, pharmacokinetic and brain-exposure analyses, extraction-based melatonin measurement, EEG/ECoG spectral analysis with simultaneous EMG recording in freely moving animals, and direct measures of neuronal injury and survival.

## 4. Materials and Methods

### 4.1. Animals

The study comprised two independent and complementary pharmacological paradigms: (I) caffeine-evoked cortical electrical activity recorded by ECoG under urethane anesthesia following surgical exposure of the cortex and (II) a pentobarbital-induced loss-of-righting-reflex test assessing sedative-hypnotic activity. The first paradigm was not intended to reproduce insomnia or physiological sleep in freely moving mice, and the second did not assess sleep architecture or sleep quality. Eighty-four male BALB/c mice (8 weeks old, 20–30 g) were obtained from the Fırat University Experimental Research Center (FUDAM, Elazığ, Türkiye). Forty-two animals were assigned to each of the two experiments, with seven mice per experimental group. Before experimentation, the animals were acclimatized to the facility for 7 days. Mice were housed four per cage on wood-shaving bedding under controlled conditions of 22 ± 2 °C and a 12:12 h light–dark cycle. Lights were switched on at 07:00 and off at 19:00; therefore, zeitgeber time 0 (ZT0) corresponded to lights on and ZT12 to lights off. Standard chow and water were provided ad libitum. All procedures were conducted in accordance with national regulations for laboratory animal care and complied with ARRIVE 2.0 guidelines. Animal health and welfare were monitored daily by trained personnel through assessment of general appearance, body condition, posture, locomotor activity, grooming, food and water intake, and signs of pain or distress.

Electrophysiological recordings were conducted using an ECoG acquisition and analysis system (AD Instruments, Castle Hill, NSW, Australia). No additional inclusion or exclusion criteria were applied during the conduct of the experiments. Exclusion criteria and data-handling rules were established a priori, specifying that animals would only be excluded in the event of severe illness, accidental death, technical failure preventing data acquisition, or inability to complete the experimental protocol. No animals met these criteria. No animals, experimental units, or recorded data points were excluded from the analyses. Therefore, all animals assigned to each experimental group were included in the final statistical analyses.

### 4.2. Caffeine-Evoked Cortical Activity Under Urethane Anaesthesia and ECoG Recordings

Animals were randomly divided into six experimental groups (n = 7 per group): (1) Control (saline + empty capsule); (2) Caffeine (7.5 mg/kg, i.p. + empty capsule; Sigma-Aldrich, St. Louis, MO, USA); (3) Caffeine + melatonin (10 mg/kg, oral); (4) Caffeine + melatonin (20 mg/kg, oral); (5) Caffeine + LMP-melatonin (10 mg/kg, oral), and (6) Caffeine + LMP-melatonin (20 mg/kg, oral). The melatonin standard was received from Sigma-Aldrich (St. Louis, MO, USA). A melt produced the proprietary LMP formulation–spray–congeal process, yielding spherical lipid microparticles of approximately 50–300 µm.

Standard melatonin, LMP-melatonin, or the corresponding empty capsule was administered orally using prefilled gelatin capsules (Bayfar, Istanbul, Türkiye), and a TORPAC oral dosing syringe (TORPAC Inc., Fairfield, NJ, USA) [13,58]. Premeasured doses of standard melatonin or LMP-melatonin were placed in empty gelatin capsules, which were then closed, loaded into the dosing syringe, and delivered directly into the stomach to ensure accurate and consistent dosing. Animals in the control and caffeine-only groups received empty capsules using the same administration procedure. Standard melatonin, LMP-melatonin, or the corresponding empty capsule was administered 45 min before the initiation of ECoG recording, 15 min before the induction of urethane anesthesia, and 60 min before caffeine or saline injection. Urethane anesthesia was induced 30 min before the initiation of ECoG recording. Following surgical preparation and electrode placement, ECoG activity was recorded continuously from 0 to 120 min. The first 15 min of the recording period served as the pre-challenge baseline. Caffeine or saline was administered intraperitoneally at minute 15 of the ECoG recording, after which recording was continued until minute 120.

Based on established stimulant-based insomnia paradigms [59], caffeine dissolved in 0.9% saline was administered intraperitoneally to induce arousal. For ECoG recordings, anesthesia was induced with urethane (1.75 g/kg, i.p.; Sigma-Aldrich (St. Louis, MO, USA) dissolved in isotonic saline 30 min before the initiation of recording. After confirmation of a stable anesthetic plane, animals were positioned in a stereotaxic frame (RWD Life Science Inc., Shenzhen, Guangdong, China), body temperature was continuously monitored and maintained with a heating pad system (RWD-69020, RWD Life Science, Shenzhen, Guangdong, China). The left cortical surface was surgically exposed, and two Ag–AgCl spherical electrodes (AD Instruments, Castle Hill, NSW, Australia) were positioned relative to bregma at 1 mm anterior and 1.5 mm lateral and at 3 mm posterior and 1.5 mm lateral, respectively.

Regional cortical electrical activity was recorded continuously for 120 min using a PowerLab data-acquisition system. Signals were sampled at 1000 Hz using LabChart 8 software (version 8.1.25) (AD Instruments, Castle Hill, NSW, Australia) [59]. The recordings were divided into eight consecutive 15 min epochs and analyzed offline. The 0–15 min epoch represented the pre-caffeine or pre-saline baseline, whereas the subsequent epochs represented post-challenge recording periods. Spike frequency was quantified using the Cycle Count function, and amplitude was calculated as the mean peak-to-peak amplitude using the Average Cyclic Height function. Cycles were identified using a two-sided height detector with maximum triggering and a minimum peak height threshold of 2.5 SD, following 5 s auto-level normalization. No additional minimum event-duration or minimum-period criterion was applied. Identical detection settings were used for all animals and treatment groups. For each mouse, spike-frequency and amplitude values were normalized to the mean value obtained during the 0–15 min pre-challenge baseline interval, which was assigned a value of 100%. All seven mice in each group contributed data at every analyzed time point, and the individual mouse was considered the experimental unit. Treatment administration was performed with knowledge of group allocation; however, offline ECoG analysis was conducted by an investigator blinded to treatment identity using coded recordings. After completion of the 120 min recording period, the mice were euthanized under urethane deep anesthesia, and blood and brain samples were collected, processed, and stored as described in Section 4.4.

### 4.3. Pentobarbital-Induced Sleep Test

An independent cohort measured sedative–hypnotic efficacy using a pentobarbital-induction sleep paradigm [60]. The mice were randomly divided into six groups as follows: (1) Control (0.9% saline), (2) Pentobarbital (42 mg/kg, i.p; Sigma-Aldrich (St. Louis, MO, USA), (3) Pentobarbital + melatonin 10 mg/kg, (4) Pentobarbital + melatonin 20 mg/kg, (5) Pentobarbital + LMP-melatonin 10 mg/kg and (6) Pentobarbital + LMP-melatonin 20 mg/kg (n = 7 per group). All animals were fasted overnight (approximately 12 h) before testing. The pentobarbital-induced sleep experiments were conducted between 13:00 and 17:00, corresponding to ZT6–ZT10 and therefore occurring entirely during the light phase. Standard or LMP-melatonin was administered orally (10 or 20 mg/kg) in a single dose using the TORPAC system [13,58]. Forty-five minutes later, pentobarbital was injected intraperitoneally at a hypnotic dose of 42 mg/kg [61]. Sleep latency was defined as the interval between pentobarbital injection and loss of the righting reflex, whereas sleep duration was defined as the interval between loss and recovery of the righting reflex. Loss of the righting reflex was considered to have occurred when a mouse placed in the supine position failed to right itself for more than 30 s. Measurements were performed by observers blinded to treatment allocation. Because saline-treated control mice did not receive pentobarbital and did not lose the righting reflex, sleep latency and sleep duration were considered undefined for this group. Accordingly, these animals were not assigned zero values and were excluded from the statistical analyses of sleep latency and duration. Animals failing to lose the righting reflex within 10 min after pentobarbital administration were predefined as nonresponders; however, no animal met this criterion, and all pentobarbital-treated mice were included in the analysis. After completion of the behavioral assessment, mice were deeply anesthetized with pentobarbital and euthanized. No serum or tissue samples from Experiment 2 were used for biochemical, qPCR, or Western blot analyses.

### 4.4. Sample Collection and Biochemical Assays

Following completion of the 120 min ECoG recording in Experiment 1, blood and brain tissues were collected, and the mice were euthanized under deep urethane anesthesia. These samples were used for all serum, qPCR, and Western blot analyses. In Experiment 2, mice were euthanized under deep anesthesia after completion of the pentobarbital-induced sleep assessment, and no samples from this cohort were included in the biochemical or molecular analyses. All terminal blood and tissue samples were collected between 14:00 and 16:00. This sampling interval corresponded to ZT7–ZT9 and was therefore within the light phase of the light/dark cycle. Blood was collected into serum-separator tubes and centrifuged at 3000 rpm for 10 min to isolate the serum. Brains were rapidly removed, and the cortex, hippocampus, and hypothalamus were dissected on ice, snap-frozen in liquid nitrogen, and stored at −80 °C until analysis.

Serum concentrations of melatonin, serotonin, and dopamine were quantified using mouse-specific ELISA kits from SunRed Biological Technology Co., Ltd. (Shanghai, China), whereas serum CRP concentration was measured using a mouse-specific ELISA kit from Elabscience Biotechnology Co., Ltd. (Wuhan, China). SunRed kits were used for melatonin (Cat. No. 201-02-0121; range, 0.8–200 ng/L; sensitivity, 0.727 ng/L), dopamine (Cat. No. 201-02-0668; range, 0.03–6 ng/mL; sensitivity, 0.03 ng/mL), and serotonin (Cat. No. 201-02-1698; range, 1–300 ng/mL; sensitivity, 0.867 ng/mL). CRP was measured using the Elabscience kit E-EL-M0053 (range, 0.03–25 ng/mL; sensitivity, 0.02 ng/mL). Serum concentrations of SOD, CAT, and GSH-Px were subsequently measured using mouse-specific SunRed ELISA kits: SOD (Cat. No. 201-02-0291; range, 0.5–100 ng/mL; sensitivity, 0.377 ng/mL), CAT (Cat. No. 201-02-1538; range, 0.75–200 ng/mL; sensitivity, 0.731 ng/mL), and GSH-Px (Cat. No. 201-02-0395; range, 1–200 ng/mL; sensitivity, 0.727 ng/mL). Because these assays quantified immunoreactive protein concentrations, the results were expressed as concentrations rather than enzymatic activities. All assays were performed according to the manufacturers’ instructions using an ELx-800 microplate reader (BioTek Instruments, Winooski, VT, USA). Manufacturer-reported intra- and inter-assay CVs were <10% and <12%, respectively. Serum serotonin and dopamine were interpreted as circulating peripheral neurochemical measures rather than direct indicators of central neurotransmission.

Serum malondialdehyde (MDA) levels, a marker of lipid peroxidation, were measured by high-performance liquid chromatography (HPLC; Shimadzu, Tokyo, Japan), as previously described with slight modifications. Serum MDA levels were determined using a thiobarbituric acid-based HPLC method with fluorescence detection. Briefly, 50 µL of undiluted serum was mixed with 400 µL of 0.1% o-phosphoric acid and 100 µL of 0.6% thiobarbituric acid. The mixture was incubated at 90 °C for 30 min and subsequently cooled on ice. The resulting MDA–TBA_2_ adduct was separated on a C18 column using 50 mmol/L potassium dihydrogen phosphate buffer (pH 6.8) and methanol (60:40, *v*/*v*) as the mobile phase. Fluorescence detection was performed at excitation and emission wavelengths of 527 and 551 nm, respectively. The data were acquired and quantified using the LC Solution software package (LabSolution LCsolution Release 1.21 Shimadzu, Tokyo, Japan) [62].

### 4.5. mRNA Expression Analyses

The mRNA expression levels of genes associated with inhibitory, serotonergic, and excitatory neurotransmission, as well as inflammatory responses, were analyzed separately in the cortex, hippocampus, and hypothalamus of each animal. Brain regions were processed and evaluated independently. The selected target genes included Gabra2, Gabbr1, Gabbr2, Htr1a, Gria1, Grin1, Grin2a, Il6, and Tnf, which were quantified using quantitative real-time PCR (RT-qPCR). Total RNA was extracted from tissue samples using the High Pure RNA Isolation Kit (Roche Diagnostics GmbH, Mannheim, Germany) according to the manufacturer’s instructions. A DNase I treatment step was included during RNA isolation to remove potential genomic DNA contamination. RNA concentration and purity were determined spectrophotometrically based on A260/A280 ratios. No separate electrophoretic assessment of RNA integrity was performed. For reverse transcription, 1 µg of total RNA was converted into complementary DNA (cDNA) in a final reaction volume of 20 µL using the Transcriptor First Strand cDNA Synthesis Kit (Roche Diagnostics GmbH, Mannheim, Germany) according to the manufacturer’s protocol. For RT-qPCR, 5 µL of cDNA was used as the template in each 20 µL reaction.

Quantitative PCR reactions were performed in a final volume of 20 µL using the LightCycler 480 SYBR Green I Master (Roche Diagnostics GmbH, Mannheim, Germany) with gene-specific primers for the target genes and Gapdh as the endogenous reference gene. The specificity of the selected primer sequences was verified using the NCBI Primer-BLAST tool, incorporating Primer3 version 2.5.0 (National Center for Biotechnology Information, Bethesda, MD, USA). Amplification was performed using a Rotor-Gene Q real-time PCR cycler equipped with a 72-well rotor (QIAGEN, Hilden, Germany). All reactions were performed in triplicate. No-template controls (NTCs) and no-reverse-transcription (No-RT) controls were included in each RT-qPCR run to monitor contamination and confirm the absence of genomic DNA amplification. The amplification protocol consisted of an initial denaturation step at 95 °C for 10 min, followed by 40 amplification cycles of denaturation at 95 °C for 15 s, annealing at 65 °C for 30 s, and extension at 72 °C for 15 s. Melting curve analysis was performed to assess amplification specificity. The stability of Gapdh as an endogenous reference gene was evaluated using the comparative Ct (ΔCt) method by comparing Ct values among experimental groups. No significant variation in Gapdh expression was detected among groups; therefore, Gapdh was used as the endogenous reference gene for normalization of target gene expression. Relative mRNA expression levels were calculated using the 2^−ΔΔCt^ method and expressed as fold changes relative to the control group. Primer sequences and amplification characteristics are presented in Table S1.

### 4.6. Western Blot Analyses

Protein levels of GABA_A_R2, GABA_B_R1, GABA_B_R2, 5-HT1A, GluA1, GluN1, GluN2A, IL-6, and TNF-α in cortex, hippocampus, and hypothalamus samples were evaluated by means of immunoblotting with respect to Mini-Protean Tetra cell (Bio-Rad Laboratories, Inc.; Hercules, CA, USA) electrophoresis followed by Pierce Power Blotter turbo blot system (Thermo Fisher Scientific, Rockford, IL, USA) [63,64]. Brain tissues obtained from all seven animals in each experimental group were processed and analyzed separately. Each experimental group included seven independent biological replicates for each brain region and protein target, and each plotted data point represented the normalized protein expression value from one individual animal. Tissue from mice was weighed at the end of the study to represent each group. Tissue protein extraction was performed as follows: tissue samples (40 mg) were homogenized in radioimmunoprecipitation (RIPA) lysis buffer containing a mixture of phosphatase-protease inhibitor and protease inhibitor (PMSF) and incubated for 30 min on ice. The lysates were centrifuged at 12,000 *g* for 40 min, and the supernatants containing the protein fraction were collected into fresh 1.5 mL Eppendorf tubes. Supernatants were subsequently combined with 1× loading buffer at a 1:1 ratio and denatured by incubation for 10 min in a water bath maintained at 95 °C. A Nanodrop/nanometer spectrophotometer (Maestro-Nano, Maestrogen Inc., Las Vegas, NV, USA) was used to measure the protein concentrations of the samples. Equal amounts of total protein from each individual sample (20 µg) were mixed with loading buffer, denatured at 95 °C for 10 min, and separated using SDS-PAGE consisting of a 12% resolving gel and a 4% stacking gel. The polyacrylamide gel and the nitrocellulose membrane were placed in direct contact for electrotransfer, placed in the cassette of the Power Blotter system with filter papers, and compressed to transfer proteins to the nitrocellulose membrane. Following transfer, membranes were blocked with 5% bovine serum albumin (BSA) in TBST for 2 h at room temperature and incubated overnight at 4 °C with the corresponding primary antibodies.

Primary antibodies against IL-6 (Cat. No. DF6087), GluR1/GluA1 (Cat. No. AF6306) GABBR1 (Cat. No. AF6520), and GABRB2 (Cat. No. DF7800) were purchased from Affinity Biosciences (Liyang, Jiangsu, China). Primary antibodies against HTR1A (Cat. No. E-AB-15447), TNF-α (Cat. No. E-AB-33121), NMDAR1/GluN1 (Cat. No. E-AB-93399), GRIN2A/GluN2A (Cat. No. E-AB-68307), GABRA2 (Cat. No. E-AB-91971), and β-actin (Cat. No. E-AB-40338) were obtained from Elabscience Biotechnology Co., Ltd. (Wuhan, Hubei, China). After primary-antibody incubation, membranes were washed and incubated for 90 min with an HRP-conjugated goat anti-rabbit IgG (H + L) secondary antibody (Cat. No. E-AB-1003; Elabscience Biotechnology Co., Ltd., Wuhan, Hubei, China). Immunoreactive bands were visualized using 0.03–0.05% diaminobenzidine prepared in 1 M Tris buffer (pH 7.4). Images were acquired under identical, non-saturating conditions, and background-corrected integrated band densities were quantified using ImageJ software (version 1.54; National Institutes of Health, Bethesda, MD, USA). Each target-protein signal was normalized to the β-actin signal obtained from the same lane.

### 4.7. Statistical Analysis

Power analysis was performed using G*Power software (version 3.1.9.2) [65]. For Experiment 1, the prespecified primary outcome was the treatment-related change in ECoG spike frequency in the caffeine-evoked arousal model, whereas for Experiment 2, the primary outcome was pentobarbital-induced sleep duration. For each experiment, an a priori sample size calculation was performed using a fixed-effects omnibus one-way ANOVA with six treatment groups. Assuming a Cohen’s f effect size of 0.65 [66], a two-sided significance level (α) of 0.05, and a statistical power of 85%, the required sample size was calculated as 42 mice per experiment, corresponding to seven animals per group. All statistical analyses were performed using GraphPad Prism (version 11.0.2; GraphPad Software, San Diego, CA, USA). Normality was evaluated using the Shapiro–Wilk test, and homogeneity of variances was assessed using Levene’s test. When these assumptions were satisfied, animal-level electrophysiological, behavioral, serum biochemical, oxidative stress, RT-PCR, and Western blot data were analyzed using one-way analysis of variance (ANOVA), followed by Tukey’s multiple-comparison post hoc test when variance homogeneity was confirmed. Because sleep latency and sleep duration were not defined for the saline control group, this group was excluded from these analyses, and comparisons were performed only among the five pentobarbital-treated groups using one-way ANOVA followed by the appropriate post hoc test. In addition, ECoG data were analyzed using two-way repeated measures ANOVA. Statistical significance was defined as *p* < 0.05.

## 5. Conclusions

LMP-melatonin produced greater modulation of caffeine-evoked cortical electrical activity under urethane anaesthesia and greater enhancement of pentobarbital-induced sedative-hypnotic responses than conventional melatonin. These effects were accompanied by more favorable changes in circulating neurochemical and oxidative-stress markers and in neurotransmitter- and inflammation-related molecular outcomes, supporting formulation-dependent differences in neuromodulatory, antioxidant, and anti-inflammatory activity. However, the present study did not directly establish enhanced bioavailability, increased central melatonin exposure, activation of specific intracellular signaling pathways, physiological sleep promotion, or neuronal protection. Physiological sleep architecture, cortical spectral power, sleep quality, and direct markers of neuronal injury were not assessed. Further studies incorporating pharmacokinetic and brain-exposure profiling, an empty LMP-matrix control, EEG/EMG-based sleep staging in freely moving animals, and direct measures of neuronal injury and survival are required to clarify the mechanisms and translational significance of these findings.

## Data Availability

The data supporting the findings (numerical source data, raw ECoG files, RT-qPCR Cq values, and uncropped original Western blot images) of this study are available from the corresponding author upon reasonable request.

## Supplementary Materials

The following supporting information can be downloaded at: https://www.mdpi.com/article/10.3390/ijms27156906/s1.

## Abbreviations

The following abbreviations are used in this manuscript:

5-HT1A	5-hydroxytryptamine receptor 1A
A1/A2A	adenosine A1 and A2A receptors
AMPA	AMPA
ANOVA	analysis of variance
CAT	catalase
cDNA	complementary DNA
CRP	C-reactive protein
ECoG	electrocorticography
EEG	electroencephalography
ELISA	enzyme-linked immunosorbent assay
EMG	electromyography
GABA	gamma-aminobutyric acid
GABA_A_R2	gamma-aminobutyric acid type A receptor subunit alpha 2
GABA_B_R1b	gamma-aminobutyric acid type B receptor subunit 1
GABA_B_R2	gamma-aminobutyric acid type B receptor subunit 2
GAPDH	glyceraldehyde-3-phosphate dehydrogenase
GPx	glutathione peroxidase
GluA1	glutamate ionotropic receptor AMPA type subunit 1
GluN1	glutamate ionotropic receptor NMDA type subunit 1
GluN2A	glutamate ionotropic receptor NMDA type subunit 2A
HClO_4_	perchloric acid
HPLC	high-performance liquid chromatography
IgG	immunoglobulin G
IL-6	interleukin-6
i.p.	intraperitoneal
KH_2_PO_4_	potassium dihydrogen phosphate
LMP	lipid-multiparticulate
MDA	malondialdehyde
mRNA	messenger RNA
MT1/MT2	melatonin receptors 1 and 2
MyD88	myeloid differentiation primary response 88
NF-κB	nuclear factor kappa B
NIH	National Institutes of Health
No-RT	no-reverse-transcription control
Nrf2	nuclear factor erythroid 2-related factor 2
NTC	no-template control
PBS	phosphate-buffered saline
PCR	polymerase chain reaction
PMSF	phenylmethylsulfonyl fluoride
REM	rapid eye movement
RNA	ribonucleic acid
RIPA	radioimmunoprecipitation assay
RT-PCR	real-time polymerase chain reaction
RT-qPCR	reverse transcription quantitative polymerase chain reaction
SD	standard deviation
SEM	standard error of the mean
SOD	superoxide dismutase
TLR4	Toll-like receptor 4
TNF-α	tumor necrosis factor-alpha
UV	ultraviolet
