# Supplementary material for: Effects of Melatonin Formulations on Caffeine-Evoked Cortical Activity and Pentobarbital-Induced Sleep in Mice
Source: Int J Mol Sci. 2026 Aug 1;27(15):6906. doi: 10.3390/ijms27156906 (PMC13466267; doi:10.3390/ijms27156906)
Supplement: Supplementary file 1 [file ijms-27-06906-s001.zip › ijms-4413991-supplementary.pdf]

Supplementary File

Effects of Melatonin Formulations on Caffeine-Evoked Cortical Activity and Pentobarbital-Induced Sleep in Mice

Nurhan Sahin[1], Mehmet Yabas[2], Besir Er[3], Fusun Erten[4], Oznur Ece Durmaz Kursun[5], Ismail Gurkan Cikim[6], Berkan Kaplan[7], Gozde Erek[1], Busra Ozmen[1], Cemal Orhan[1], Ertugrul Kilic[8], Kazim Sahin[1]

[1] Department of Animal Nutrition, Faculty of Veterinary Medicine, Firat University, Elazig 23119, Türkiye; nsahin@firat.edu.tr (N.S.); gerek@firat.edu.tr (G.E.); busraagzikucuk01@gmail.com (B.O.); corhan@firat.edu.tr (C.O.); ksahin@firat.edu.tr (K.S.)
[2] Department of Immunology, Faculty of Medicine, Malatya Turgut Ozal University, Malatya 44210, Türkiye; mehmetyabas@ozal.edu.tr (M.Y.)
[3] Department of Medical Services and Techniques, Vocational School of Health Services, Firat University, Elazig 23119, Türkiye; ber@firat.edu.tr (B.E.)
[4] Department of Veterinary Science, Pertek Sakine Genç Vocational School, Munzur University, Tunceli 62500, Türkiye; fusunerten@munzur.edu.tr (F.E.)
[5] Department of Pharmacology, Faculty of Pharmacy, Firat University, Elazig 23119, Türkiye; oedkursun@firat.edu.tr (O.E.D.K.)
[6] Department of Biochemistry, Faculty of Medicine, Kahramanmaras Sutcu Imam University, Kahramanmaras 46040, Türkiye; ismailgurkancikim@ksu.edu.tr (I.G.C.)
[7] Department of Neurology, Servet Unsal Medical Center, Ankara 06200, Türkiye; kaplanberkan@hacettepe.edu.tr (B.K.)
[8] Department of Physiology, Faculty of Medicine, Istanbul Medeniyet University 34700, Türkiye; ertugrul.kilic@medeniyet.edu.tr (E.K.)

* Correspondence: Dr. Nurhan Sahin, Department of Animal Nutrition, Faculty of Veterinary Medicine, Firat University, Elazig, 23119, Türkiye. E-mail: nsahin@firat.edu.tr

Hippocampus	Hypothalamus	Cortex
A) $GABA_AR2$<br>~ 55 kDa<br>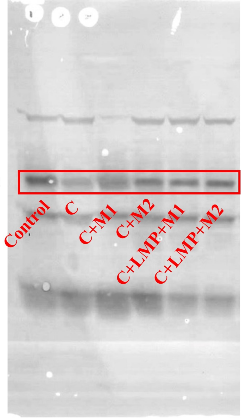	A) $GABA_AR2$<br>~ 55 kDa<br>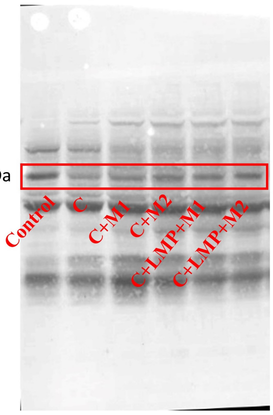	A) $GABA_AR2$<br>~ 55 kDa<br>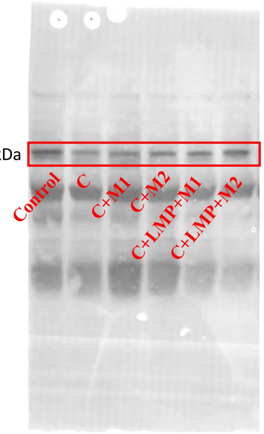
B) $GABA_BR1$<br>~ 100 kDa<br>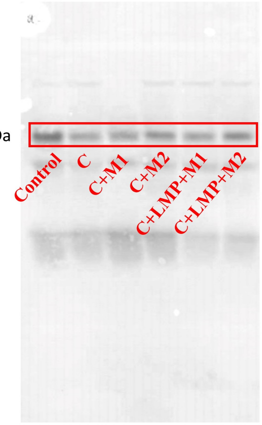	B) $GABA_BR1$<br>~ 100 kDa<br>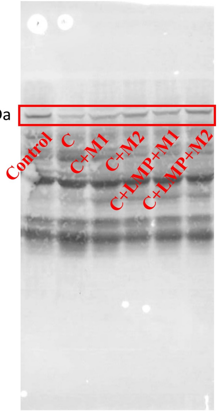	B) $GABA_BR1$<br>~ 100 kDa<br>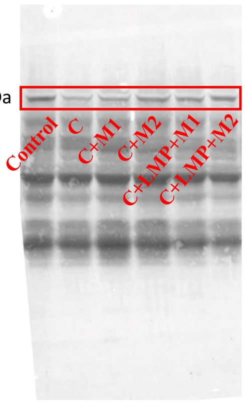
C) $GABA_BR2$<br>~ 105 kDa<br>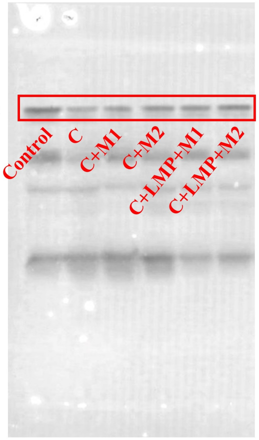	C) $GABA_BR2$<br>~ 105 kDa<br>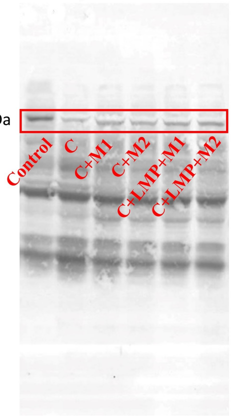	C) $GABA_BR2$<br>~ 105 kDa<br>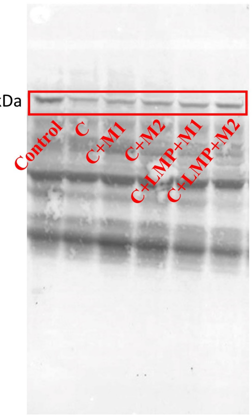

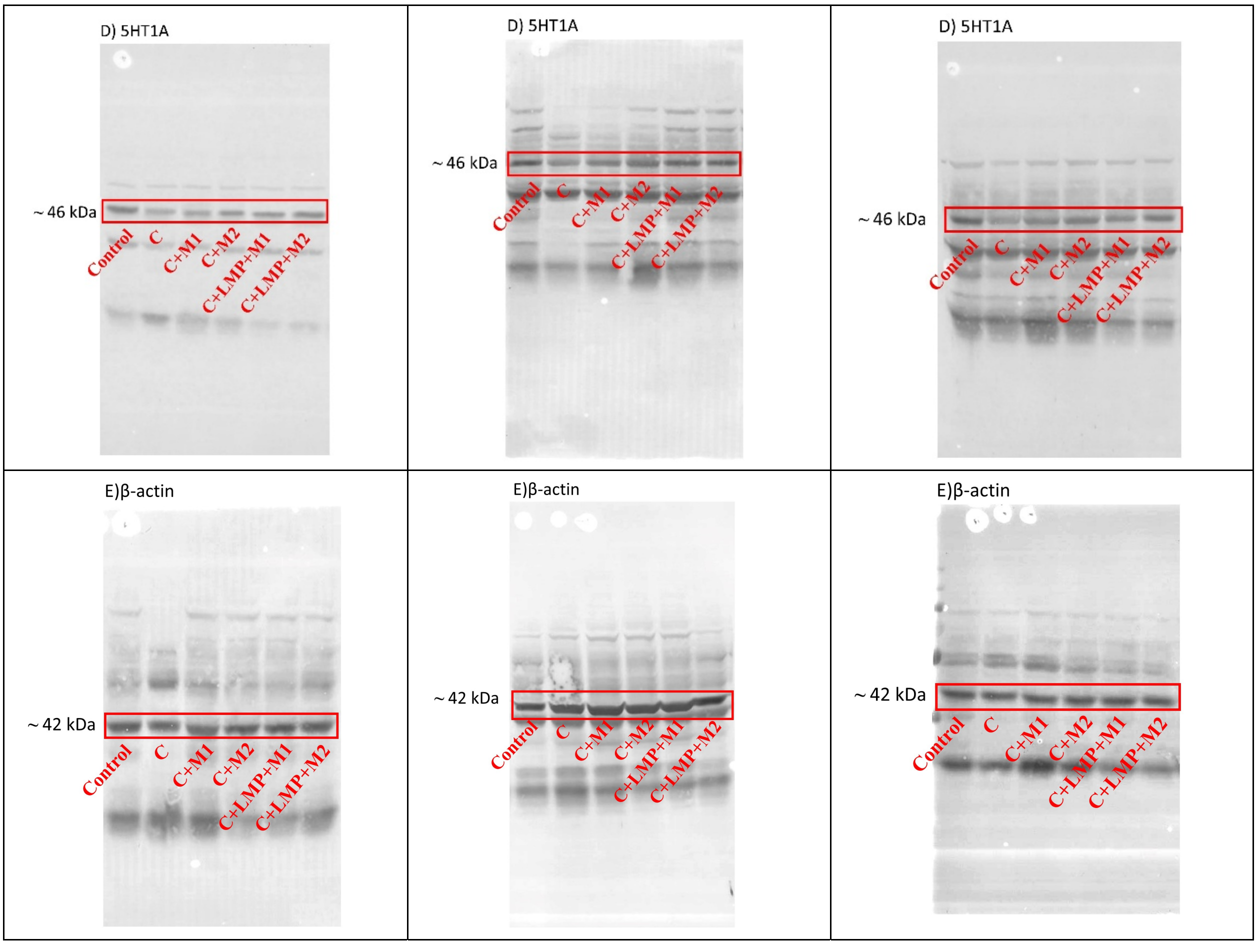

Figure S1. Full immunoblots related to Figure 5 in the main text. Representative full-length immunoblots showing the protein expression of GABA$_A$R2 (A), GABA$_B$R1 (B), GABA$_B$R2 (C), and 5-HT1A (D) and (E) β-actin. Data are expressed in hippocampus, hypothalamus, and cortex regions. Densitometric analysis of band intensities was normalized to β-actin to confirm equal protein loading and expressed relative to the control group. Red dotted rectangles indicate the bands quantified in Figure 5 of the main text. Molecular weight (M.W.) markers are shown in kilodaltons (kDa). Groups: Control (oral placebo + i.p. saline), C (caffeine alone, 7.5 mg/kg, i.p.), C+M1 (caffeine + standard melatonin, 10 mg/kg, oral), C+M2 (caffeine + standard melatonin, 20 mg/kg, oral), C+LMP-M1 (caffeine + LMP-melatonin, 10 mg/kg, oral), C+LMP-M2 (caffeine + LMP-melatonin, 20 mg/kg, oral).

Hippocampus	Hypothalamus	Cortex
A) GluA1 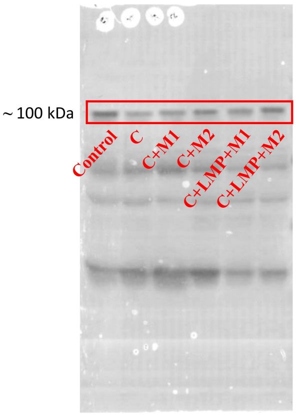	A) GluA1 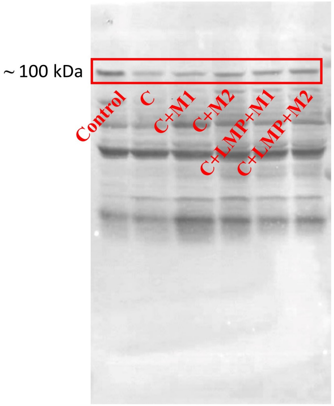	A) GluA1 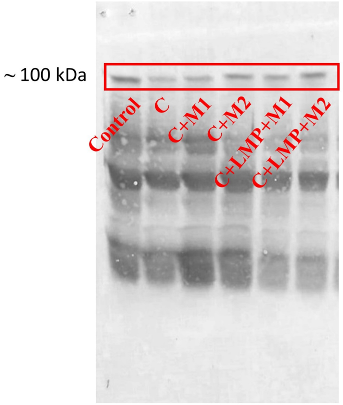
B) GluN1 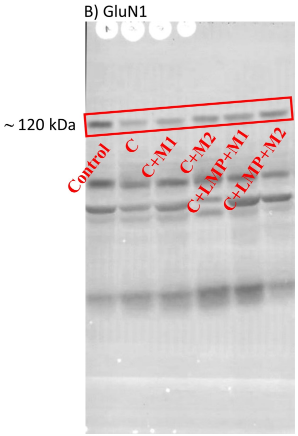	B) GluN1 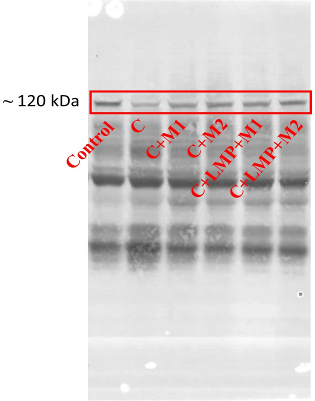	B) GluN1 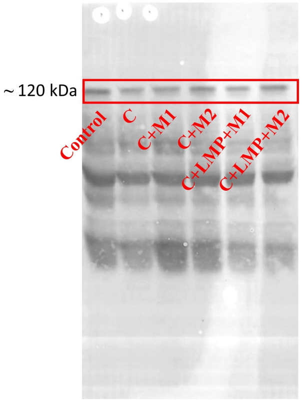
C) GluN2A 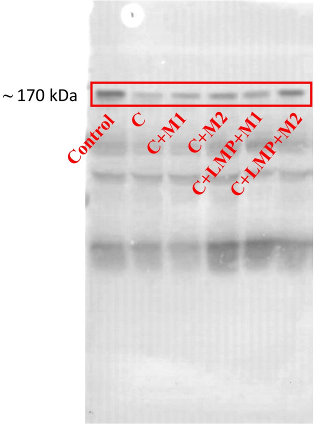	C) GluN2A 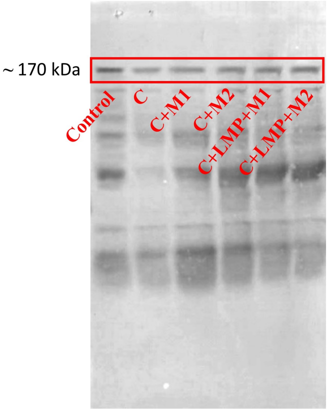	C) GluN2A 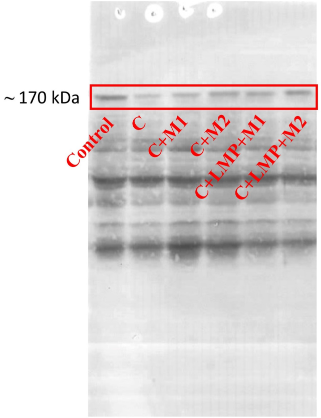

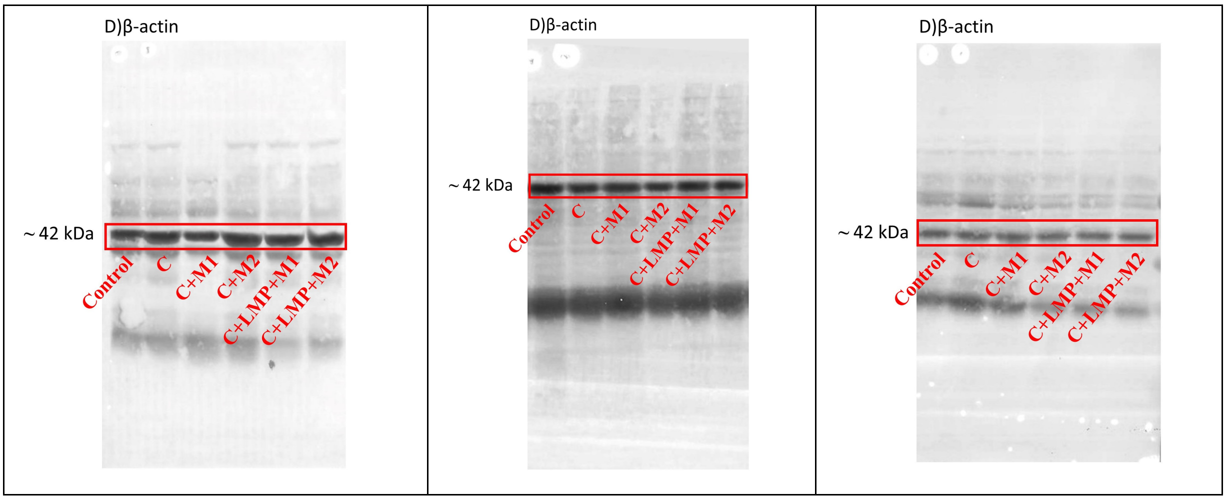

Figure S2. Full immunoblots related to Figure 7 in the main text. Representative full-length immunoblots showing the protein expression of GluA1 (A), GluN1 (B), and GluN2A (C), and (D) β-actin. Densitometric analysis of band intensities was normalized to β-actin to confirm equal protein loading and expressed relative to the control group. Red dotted rectangles indicate the bands quantified in Figure 7 of the main text. Molecular weight (M.W.) markers are shown in kilodaltons (kDa). Groups: Control (oral placebo + i.p. saline), C (caffeine alone, 7.5 mg/kg, i.p.), C+M1 (caffeine + standard melatonin, 10 mg/kg, oral), C+M2 (caffeine + standard melatonin, 20 mg/kg, oral), C+LMP-M1 (caffeine + LMP-melatonin, 10 mg/kg, oral), C+LMP-M2 (caffeine + LMP-melatonin, 20 mg/kg, oral).

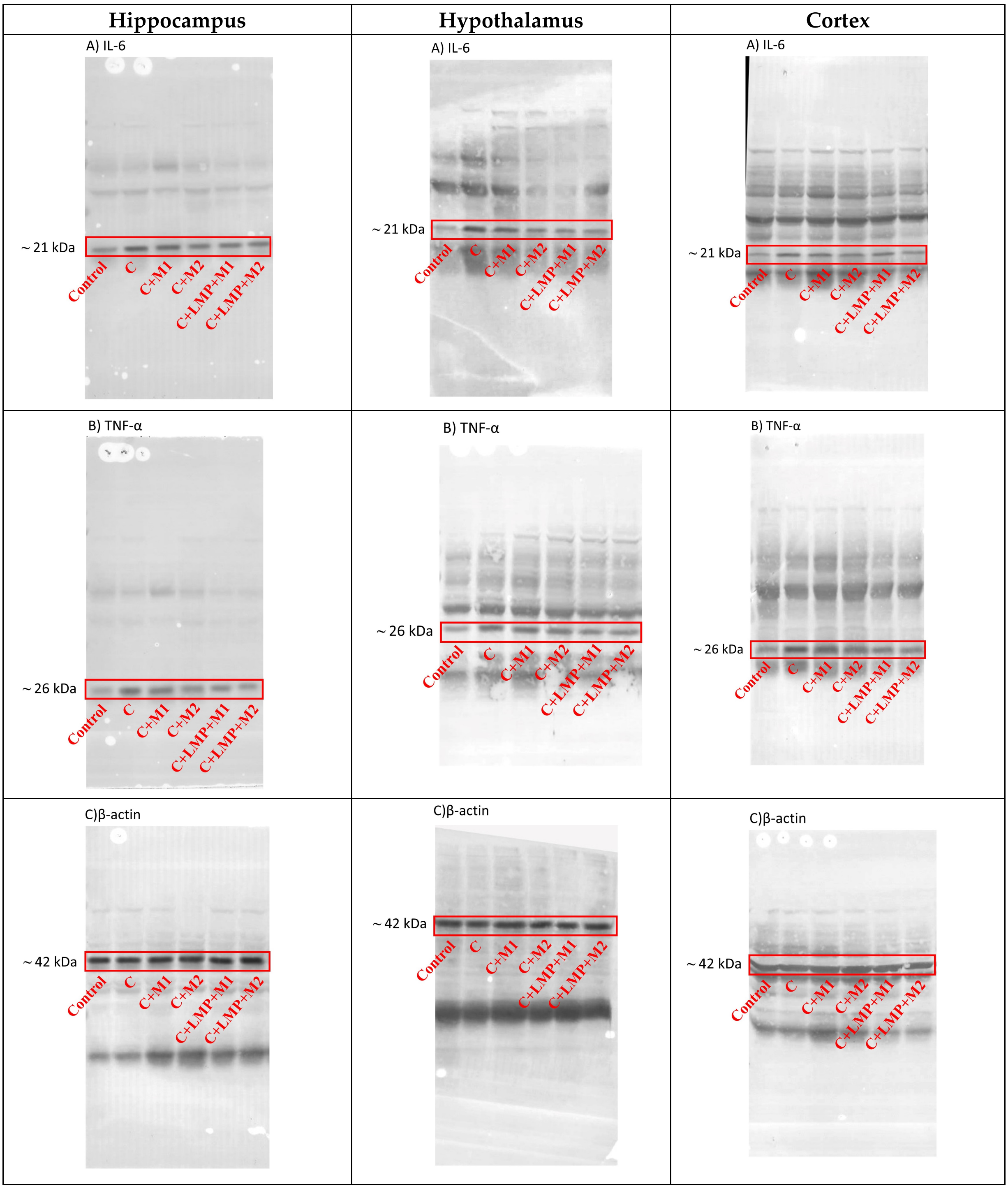

Figure S3. Full immunoblots related to Figure 9 in the main text. Representative full-length immunoblots showing the protein expression of IL-6 (A) and TNF-α (B), and (C) β-actin. Densitometric analysis of band intensities was normalized to β-actin to confirm equal protein loading and expressed relative to the control group. Red dotted rectangles indicate the bands quantified in Figure 9 of the main text. Molecular weight (M.W.) markers are shown in kilodaltons

(kDa). Groups: Control (oral placebo + i.p. saline), C (caffeine alone, 7.5 mg/kg, i.p.), C+M1 (caffeine + standard melatonin, 10 mg/kg, oral), C+M2 (caffeine + standard melatonin, 20 mg/kg, oral), C+LMP-M1 (caffeine + LMP-melatonin, 10 mg/kg, oral), C+LMP-M2 (caffeine + LMP-melatonin, 20 mg/kg, oral).

Table S1. PCR primer sequences.

Gene	RefSeq accession	Forward primer	Reverse primer	Amplicon size (bp)
Gabra2	NM_008066.5	GCTGTTCAGCTTTGGCAACA	TTAGCCAGCACCAACCTGAC	111
Gabbr1	NM_019439.4	CGGGTGGATTTCCGATGTGA	TGTGGCGTTCGATTCACCTG	116
Gabbr2	NM_001081141.2	GCGTCAAAAAGCTCAAGGGG	GCTCTCCTCAAAGGCACAAC	98
Htr1a	NM_008308.5	GCTGGTCTGGGAAAGTTGGA	AGAAAGTGGGTGCTGTGCAT	106
Gria1	NM_008165.4	AAAACTGAACGAGCAGGGGC	CACTGGTCTTGTCCTTGGAGT	104
Grin1	NM_008169.3	GAGCGTGAGTCCAAGGCAG	TGCAGAAAGGATGATGACCCG	114
Grin2a	NM_008170.4	GAGCGGGTACATCTTTGCCA	CTTCCATCTCACCGTCACCA	116
Il6	NM_031168.2	GTGGAAATGAGAAAAGAGTTGTGC	TCCAGTTTGGTAGCATCCATCA	123
Tnf	NM_013693.3	CCCACGTCGTAGCAAACCA	TGTCTTTGAGATCCATGCCGT	96
Gapdh	NM_008084.4	AGGTCGGTGTGAACGGATTT	ATGAAGGGGTCGTTGATGGC	100
